# Quantifying Cortical Maturational Aspects During Different Vigilance States in Preterm Infants by Advanced EEG Analysis

**DOI:** 10.1111/jsr.70308

**Published:** 2026-02-08

**Authors:** Gaia Burlando, Sara Uccella, Valentina Marazzotta, Sheng H. Wang, J. Matias Palva, Monica Roascio, Andrea Rossi, Luca Antonio Ramenghi, Lino Nobili, Gabriele Arnulfo

**Affiliations:** ^1^ Department of Informatics, Bioengineering, Robotics and Systems Engineering University of Genoa Genoa Italy; ^2^ Department of Neurosciences, Rehabilitation, Ophthalmology, Genetics, and Maternal and Child Health University of Genoa Genoa Italy; ^3^ Child Neuropsychiatry Unit, IRCCS Istituto Giannina Gaslini Genoa Italy; ^4^ Department of Neuroscience and Biomedical Engineering Aalto University Espoo Finland; ^5^ Neuroscience Center Helsinki Institute of Life Science (HiLIFE), University of Helsinki Helsinki Finland; ^6^ CEA, NeuroSpin Université Paris‐Saclay Gif‐sur‐Yvette France; ^7^ MIND Team Inria Palaiseau France; ^8^ Jack H. Miller Magnetoencephalography Center, Helen DeVos Children's Hospital Grand Rapids Michigan USA; ^9^ Centre for Cognitive Neuroimaging, School of Psychology and Neuroscience University of Glasgow Glasgow UK; ^10^ Neuroradiology Unit, IRCCS Istituto Giannina Gaslini Genoa Italy; ^11^ Neonatology Unit, IRCCS Istituto Giannina Gaslini Genoa Italy

**Keywords:** cortical maturation, criticality, polysomnography, prematurity

## Abstract

Preterm birth is a significant risk factor for atypical neurodevelopment, yet early electrophysiological markers of brain maturation are still lacking. Non‐invasive electroencephalographic (EEG) monitoring of cortical maturation in these patients holds promise as a tool for neurodevelopmental prediction. However, its clinical application is limited by technical challenges in maintaining stable, long‐term electrode placement on very small neonate scalps and by the highly specialised, multi‐level expertise required to care for these fragile patients. Using video‐polysomnographic EEG recordings in very low birth weight (VLBW, < 1500 g) preterm infants, we characterised large‐scale neuronal dynamics during distinct vigilance states and tested whether they could serve as indicators of early cortical maturation. We analysed EEG recordings obtained at 33.9 ± 1.4 weeks postmenstrual age (PMA), during active sleep (AS), sleep onset active sleep (SOAS), quiet sleep (QS), and quiet wakefulness (QW). For each vigilance state, we assessed large‐scale neuronal dynamics in terms of phase synchronisation, neuronal bistability, and local phase‐amplitude coupling (PAC), both globally and separately for anterior and posterior regions, and correlated them with PMA. We found that phase synchronisation peaked in the δ band during QS and in the θ band during more active states (QW, SOAS, AS). δ‐band bistability was lower in posterior regions across all states, while δ‐PAC was lower posteriorly during sleep but reversed during wakefulness. Also, bistability and PAC decreased with advancing PMA. These findings suggest that vigilance‐state‐dependent neuronal dynamics capture aspects of early cortical maturation—even with low‐density EEG cap—offering novel candidate biomarkers to monitor neurodevelopment in infants born preterm.

## 1 Introduction

Preterm birth, defined as delivery before 37 weeks of gestation, occurs in approximately 10% of live births worldwide and is associated with a heightened risk of adverse neurodevelopmental outcomes, even in the absence of overt brain injury (Marlow et al. 2021; Perin et al. 2022; Uccella et al. 2023; Malova et al. 2024). This increased risk reflects the interruption of key neurodevelopmental processes that normally occur in utero during the third trimester, such as neurogenesis, neuronal migration, synaptogenesis, and the establishment of cortico‐cortical and cortico‐subcortical connections (McQuillen and Ferriero 2005; Uylings 2006; Dubois et al. 2014; Uccella et al. 2024). During this sensitive developmental window, cortical and subcortical structures undergo progressive maturation, leading to the formation of both long‐ and short‐range networks which strengthen following spatially organised patterns (e.g., posterior‐to‐anterior). These changes are reflected in the brain's emerging functional activity (Ramenghi et al. 2007; Thomason et al. 2013, 2014; Jakab et al. 2014; van den Heuvel and Thomason 2016). Accordingly, the early postnatal period in preterm newborns represents a crucial window to evaluate and refine methods for assessing how brain maturation advances outside the womb, especially when preterm birth occurs at the earliest gestational ages. Quantitative methods for assessing brain activity at this early stage are crucial for identifying early biomarkers of neurodevelopment and understanding their potential correlation with long‐term outcomes.

Electrophysiological activity, as captured through electroencephalographic (EEG) recordings, reflects these maturational processes and can serve as a non‐invasive readout of developing network function. However, electrophysiological brain activity is neither static nor homogeneous: it fluctuates due to multiple factors, including vigilance state, particularly sleep, which is the dominant condition during this period of life. In fact, sleep occupies more than two‐thirds of the total time in preterm neonates (Roffwarg et al. 1966; Uccella et al. 2025) and it actively contributes to brain maturation (Frank 2017; Blumberg et al. 2022). Sleep itself is not a uniform phenomenon even at early developmental stages, with an alternation of distinct vigilance states. These include active sleep (AS)—the precursor of adult REM sleep—characterised by intermittent eye movements and continuous EEG activity, typically occurring after quiet sleep. Sleep onset active sleep (SOAS) is recognised when infants fall asleep from wakefulness, often showing mixed EEG patterns and low levels of movement (André et al. 2010; Grigg‐Damberger 2016). Quiet sleep (QS) is the precursor of adult NREM sleep, marked by minimal movements, presence of muscular tone, and discontinuous EEG (tracé alternant). Finally, quiet wakefulness (QW) is a calm, awake state with open eyes and low muscular activity—very difficult to recognise in the absence of appropriate video monitoring. Each of these states is associated with distinct patterns of neuronal oscillations and brain connectivity (Vanhatalo and Kaila 2006; Tokariev et al. 2016; Tokariev et al. 2019; Yrjölä et al. 2022; Shiraki et al. 2024).

Appearance, structure, and regulation of these stages follow specific developmental patterns, paralleling the maturation of underlying neural circuits (André et al. 2010; Blumberg et al. 2020; Grigg‐Damberger 2016; Uccella et al. 2024). This close link between electrophysiological activity, vigilance state organisation, and neurodevelopment suggests that analysing EEG across different vigilance states could yield critical insights into brain maturation. This study characterises state‐specific EEG biomarkers of cortical maturation in very low birth weight (VLBW) preterm infants at low neurological risk.

To interpret large‐scale neuronal dynamics within a unified theoretical perspective, we adopted the brain criticality framework. This framework conceptualises brain activity as operating near a transition point between hypo‐ and hyper‐synchronised states, a regime thought to support both stability and flexibility of neural dynamics (Haldeman and Beggs 2005; Chialvo 2010; Cocchi et al. 2017). In this state, brain activity exhibits hallmarks of critical systems, including scale‐invariance and non‐linear transitions, enabling efficient information processing (Kinouchi and Copelli 2006; Lotfi et al. 2021). Dysregulation of this balance has been implicated in developmental and pathological conditions, including epilepsy (Meisel et al. 2012; Wang et al. 2023, 2024; Burlando et al. 2025). Based on this framework, we focused our analyses on three intertwined EEG‐derived features that reflect key aspects of neuronal dynamics: (a) phase synchronisation, measuring coordination of oscillatory activity across regions (Buzsáki and Draguhn 2004; Fries 2005; Palva et al. 2005, 2018); (b) local bistability, quantifying the brain's tendency to erratically jump between more and less synchronised states near criticality (Freyer et al. 2009; Wang et al. 2023); and (c) cross‐frequency coupling, which quantifies the hierarchical coordination of activity across timescales (Canolty and Knight 2010; Palva and Palva 2018; Siebenhühner et al. 2020).

We hypothesised that network‐level EEG dynamics reflect age‐ and state‐dependent aspects of cortical maturation in preterm infants. We analysed phase synchronisation, neuronal bistability, and local cross‐frequency coupling across vigilance states using EEG data from a unique dataset of preterm infants at low neurological risk. We tested whether these metrics capture state‐specific patterns relevant to early brain development and how they vary with postmenstrual age.

Our findings provide novel quantitative indices of early brain development, which may serve as potential prognostic biomarkers for neurodevelopmental outcomes.

## 2 Methods

### 2.1 Patients and Data Acquisition

This observational study was part of a larger cohort study on preterm infants born with a very low birth weight (VLBW) approved by the Local Ethical Review Board (CERL number approval 0028224/21 of 6/10/2021). It was conducted in accordance with the Declaration of Helsinki. All data were recorded at the Neonatal Intensive Care Unit of the Gaslini Children's Hospital. Parents provided informed consent for the study. Subjects were recorded once they were hemodynamically stable and free from sedation. The study included only VLBW infants who underwent prolonged full‐channel polysomnography between 30 and 36 weeks of PMA, allowing us to assess maturational changes during this critical period in a sufficient period of artefact‐free recording.

Twenty‐one VLBW preterm infants were included. All infants had an appropriate head circumference for gestational age (Bertino et al. 2010) and showed no brain lesions on routine cranial ultrasound; minor brain lesions were later identified in 10 infants using MRI at term‐equivalent age (Uccella et al. 2023; Malova et al. 2024). Infants with moderate or severe brain lesions were excluded from the analyses. Detailed demographic and clinical characteristics are provided in Table 1.

**TABLE 1:** Demographic and clinical characteristics of the two subgroups included in the study.

	Non‐lesion (*n* = 11)	Lesion (*n* = 10)
Gestational age (weeks)	31.0 ± 2.2	30.6 ± 2.2
Postmenstrual age (weeks)	33.0 ± 1.6	34.4 ± 1.1
Birth weight (g)	1312.3 ± 190.2	1319.5 ± 302.4
Sex (M/F)	4/7	3/7
Apgar 1′	6.1 ± 2.0 (*n* = 8)	6.7 ± 1.6
Apgar 5′	8.3 ± 1.1 (*n* = 9)	8.2 ± 0.4

*Note:* The non‐lesion group includes infants without detectable brain lesions, while the lesion group includes infants with minor perinatal brain lesions. Values are reported as mean ± SD; sample sizes for Apgar scores reflect available data.

### 2.2 Signal Preprocessing

All subjects underwent a video‐polysomnography recording using a Brain QUICK (Micromed) system. Scalp cortical activity was measured using a full‐band EEG amplifier (FbEEG, SD Plus Flexi Clinic Micromed). We used non‐polarizable Sintered Ag/AgCl cup electrodes (5 mm diameter, 19 mm^2^ recording area) and secured them with electroconductive paste. Once the right and left parasagittal and lateral longitudinal lines were outlined, eight active surface electrodes were placed according to the modified 10/20 system for neonates. The reference electrode was placed on Fz, away from the bregmatic fontanelle, and the ground electrode on Pz.

The data were then selected to retain 8 monopolar EEG channels (10–20 International System), excluding EMG, ECG, and EOG, acquiring bandpass signals (0.15–134 Hz) at a sampling rate of 512 Hz.

**FIGURE 1:**
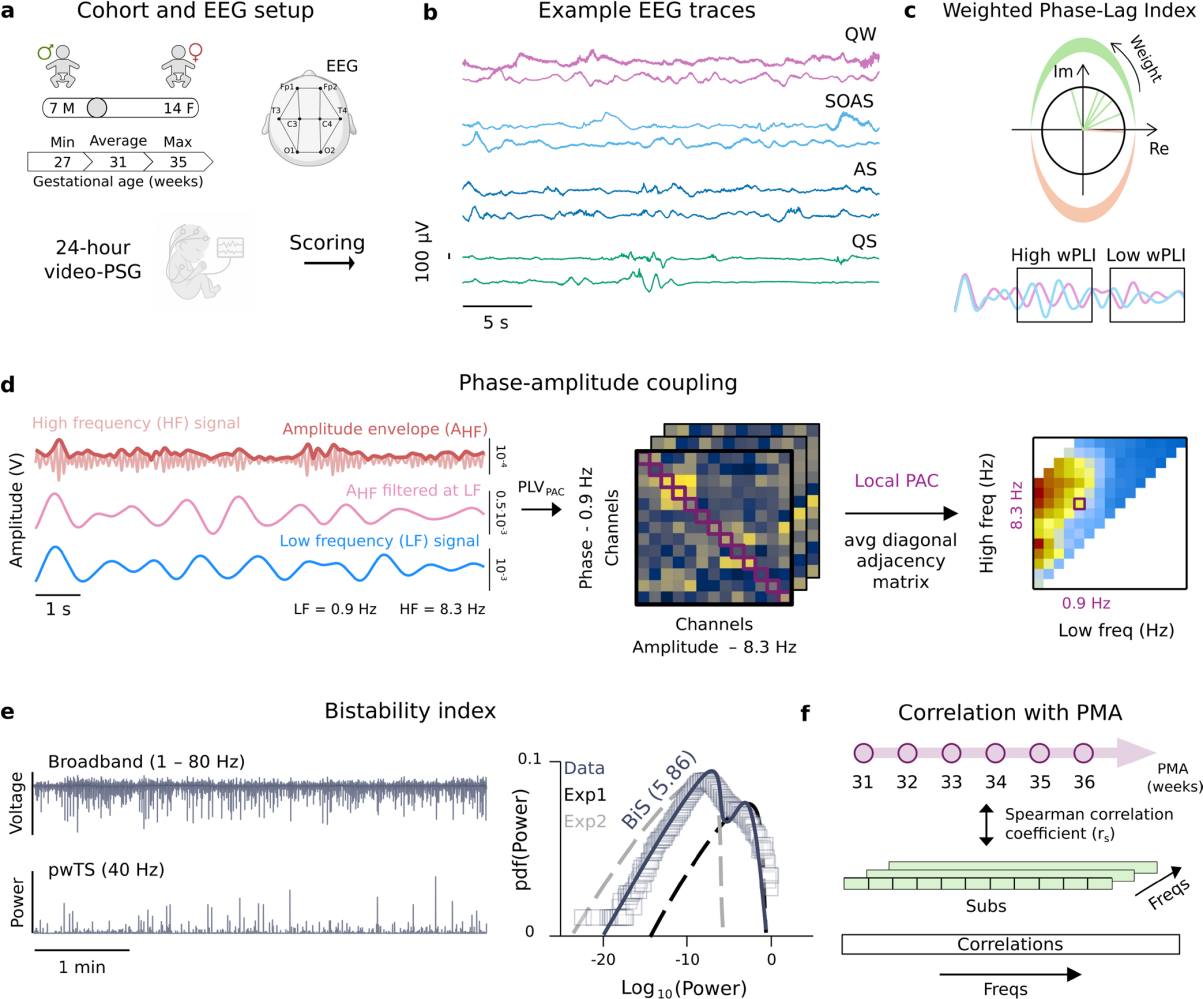
Overview of the study design and analyses. (a) Subject demographic is shown, including gestational age and recording details. (b) Each recording was classified into four vigilance states: Quiet wakefulness (QW), sleep onset active sleep (SOAS), active sleep (AS), and quiet sleep (QS), and five‐minute epochs are shown as exemplary traces for each state. (c) The weighted phase‐lag index (wPLI) was calculated to quantify phase synchronisation between pairs of bipolar‐references EEG channels. The example illustrates two time series showing an initial period of high wPLI (phase synchronised) followed by low wPLI (desynchronized). (d) Phase‐amplitude coupling (PAC) analysis was performed by filtering a broadband signal with a low‐frequency (LF) Morlet wavelet and another signal with a high‐frequency (HF) Morlet wavelet. The envelope of the HF signal was extracted, filtered at the LF, and the phase locking value (PLV) was computed between the two signals. This results in subject‐specific adjacency matrices for each vigilance state, and local PAC values were averaged across channels to obtain a single value per LF‐HF pair. (e) Left: Bipolar‐referenced broadband (BB) and its 40‐Hz narrowband power time series (pwTS). Right: A bi‐exponential fit is applied on the probability density function (pdf) to extract the bistability index (BiS). (f) Spearman correlations between PMA and each metric (wPLI, BiS, and nPAC) were computed across frequencies, producing spectra of correlation coefficients reflecting age‐dependent cortical network changes.

We excluded all harmonics of 50‐Hz line noise using a series of notch FIR filters with a 1 Hz band‐stop width. We employed a bipolar referencing scheme by pairing neighbouring electrode contacts while excluding a common reference (Figure 1a). After this step, the channels for each subject encompassed the following 13 bipolar derivations: Fp1–C3, C3–O1, Fp1–T3, T3–O1, T3–C3, Fp2–C4, C4–O2, Fp2–T4, T4–O2, C4–T4, Fp1–Fp2, C3–C4, O1–O2. This bipolar scheme is currently employed in clinical practice at the reference centre, and it is commonly adopted in neonatal EEG recordings (Stevenson et al. 2019). Given the limited number of available channels, this scheme was also adopted to minimise the impact of artefacts, even though it constrains spatial resolution and limits the characterisation of large‐scale network organisation (Tokariev et al. 2012).

The EEG recordings were manually scored by clinical experts. Quiet wakefulness (QW), sleep onset active sleep (SOAS), active sleep (AS), and quiet sleep (QS) were identified and segmented into 30‐s epochs, as recommended by the American Academy of Sleep Medicine (Grigg‐Damberger 2016).

Of note, SOAS and AS were here considered as separate behavioural states and thus treated separately in light of their electrographical differences, but also for the fact that the time spent in SOAS, more than AS, correlates with poor neurological outcomes, thus suggesting a differential role in regulating cortical development (Uccella et al. 2025).

For each subject, carefully selected artefact‐free epochs were analysed, yielding an average duration for each subject of: 8.1 ± 1.6 min (mean ± SD) for QW; 7.9 ± 1.7 min for SOAS; 7.8 ± 2.0 min for AS; and 12.0 ± 2.3 min for QS.

### 2.3 Spectral Power Estimates

To evaluate the power spectral characteristics of EEG signals across the four vigilance states in preterm infants, we calculated the power spectral density (PSD) for all bipolar channels using Welch's method (Hamming window), applied within the frequency range of 0.25–40 Hz with a resolution of 0.25 Hz. The spectra were then averaged across subjects within the same sleep stage.

### 2.4 Phase Synchrony Estimates

We estimated interareal phase interactions at the individual subject level using the weighted phase‐lag index (wPLI) (Vinck et al. 2011). This measure is computed between signals from different EEG channels and is often used to help mitigate the potential influence of volume conduction in neuronal oscillation analysis (Vinck et al. 2011; Palva et al. 2018). wPLI ranges from 0–1, where a higher value indicates stronger phase synchronisation, implying that the phases of the two signals tend to align more consistently over time. First, we applied a time‐frequency decomposition by Morlet‐wavelet filtering the data into narrow‐band time series for 50 frequencies, ranging from 0.2–40 Hz (no cycles = 7.5), logarithmically spaced (Tallon‐Baudry et al. 1996). Then, wPLI was computed as:

$$\text{wPLI}=\frac{|E\left\{I\left\{X\right\}\right\}|}{E\left\{I\left\{X\right\}\right\}}=\frac{|E\left\{I\left\{X\right\}|\cdot \mathrm{sgn}\left(I\left\{X\right\}\right)\right\}|}{E\left\{I\left\{X\right\}\right\}}$$

where $I\left\{X\right\}$ represents the imaginary part and X is the cross‐spectrum between the two EEG time series.

### 2.5 Cross‐Frequency Coupling of Slow and Fast Rhythms

Cross‐frequency interaction was assessed by calculating phase‐amplitude coupling (PAC). We quantified PAC using the phase locking value (PLV), which measures the consistency of the phase relationship between two frequency bands across different time points, providing an estimate of the coupling strength (Vanhatalo et al. 2004). We measured the PAC‐PLV to investigate the modulation of higher‐frequency (HF) oscillation amplitudes by the phase of lower‐frequency (LF) oscillations, separately for each vigilance state. The strength of PAC was quantified as:

$$\mathrm{PL}V_{\mathrm{PAC},a,b}=\frac{1}{N}\left|\sum_{n=1}^{N}e^{i\cdot \left(\theta_{a,\mathrm{LF}}-\theta_{b,\mathrm{HF},\mathrm{LF}}\right)}\right|$$

where $\theta_{b,\mathrm{HF},\mathrm{LF}}$ is the phase of the amplitude envelope of the HF signal filtered with a Morlet filter at LF. Local PAC was obtained where a=b.

As low frequencies, we used the same 50 as in the phase synchrony estimates, while the high frequencies were computed using 19 slow‐to‐fast ratios ranging from 1:2–1:100. The PAC matrices, initially structured in a low frequency‐to‐ratio format, were interpolated and mapped into a low frequency‐to‐high frequency space to improve readability.

Results show normalised PAC (nPAC), where nPAC = PLV_PAC,observed_/PLV_PAC,surrogate_, so that nPAC > 1 indicates PAC above the null hypothesis level.

### 2.6 Bistability of Neuronal Oscillations Estimates

We estimated bistability in narrow‐band neuronal oscillations using the bistability index (BiS) (Freyer et al. 2011; Wang et al. 2023). The process involves fitting the observed probability distribution of the narrow‐band power time series with both a single exponential and a bi‐exponential model. Subsequently, BiS is determined by comparing these two models using the Bayesian information criterion. A BiS close to zero means that the single‐exponential model is a more likely model for the observed time series, whereas for a BiS greater than 0, the most likely model for the observed time series is the bi‐exponential model. We applied Morlet wavelet filtering to extract narrow‐band time series for the same 50 frequencies as before and then computed BiS separately for each vigilance state.

### 2.7 Statistical Analysis

Statistical analyses were performed using Python 3.11. When comparing metrics across the four vigilance states, we used the Kruskal‐Wallis test followed by Benjamini‐Hochberg (BH) correction for multiple comparisons along the frequency axis. In this case, to assess the effect size for comparisons involving more than two groups (i.e., for the four vigilance states), we computed the η^2^ as follows:

$$\eta_{H}^{2}=\frac{H-k+1}{n-k}$$

where H is the value obtained in the Kruskal‐Wallis test, k is the number of groups (i.e., the four vigilance states), and n is the total number of observations. The eta‐squared ranges from 0–1, with values typically classified as: 0.01 ≤ η^2^ ≤ 0.06 (small effect), 0.06 ≤ η^2^ ≤ 0.14 (moderate effect), and η^2^ ≥ 0.14 (large effect) (B. H. Cohen 2008).

For pairwise comparisons (i.e., lesion vs. non‐lesion and anterior vs. posterior derivations), we applied the Wilcoxon rank‐sum test, also with BH correction. To quantify the effect size for these two‐group comparisons, we calculated the rank‐biserial correlation (RBC) (Kerby 2014). RBC values were interpreted as follows: 0.1 ≤ RBC ≤ 0.3 (small effect), 0.3 ≤ RBC ≤ 0.5 (medium effect), and RBC ≥ 0.5 (large effect) (J. Cohen 2013; Tomczak and Tomczak 2014).

### 2.8 Correlation Estimates With PMA

To explore the dynamic changes in neuronal networks driven by cortical maturation, we analysed the correlations between phase synchronisation, bistability, and cross‐frequency coupling with PMA. To assess the predictive value of BiS, wPLI, and nPAC on PMA, we employed three separate linear mixed‐effects models (LMMs) (Raudenbush and Bryk 2002). These models were fitted using restricted maximum likelihood estimation (REML), with each metric as the dependent variable and PMA, sex, and sleep stage (SOAS as the reference stage) as fixed effects. Frequency bands were incorporated as random effects to account for repeated measures.

To examine frequency‐specific effects, post hoc Spearman rank correlations were computed separately for each frequency. Confidence intervals were estimated via permutation of PMA values, and multiple comparisons across frequencies were corrected using the maximum and minimum bounds of the permuted distributions.

### 2.9 Post Hoc Power Assessment

Post hoc power was estimated using the Fisher z transformation for correlation coefficients. The observed correlation r was transformed as:

$$z_{r}=\frac{1}{2}\ln\left(\frac{1+r}{1-r}\right)$$

With standard error

$$\sigma_{z}=\frac{1}{\sqrt{n-3}}$$

For a two‐tailed test at α=0.05, power was computed as

$$\text{Power}=1-\Phi \left(z_{\text{crit}}-\mu \right)+\Phi \left(-z_{\text{crit}}-\mu \right)$$

where $\mu =z_{r}/\sigma_{z}$ and $z_{\text{crit}}=1.96$ is the critical value of the standard normal distribution for a two‐sided test at *p* < 0.05.

For our cohort of 21 subjects, we calculated power for the minimum and maximum observed significant correlations (*r* = 0.5 and *r* = 0.85), obtaining a power of 0.64 and 0.99, respectively.

## 3 Results

### 3.1 Neuronal Oscillatory Dynamics Are Preserved in Infants Born With Minor Brain Lesions

To assess whether brain lesions influence neuronal dynamics, we compared phase synchronisation, bistability, and cross‐frequency PAC between infants with (i.e., lesion) and without (i.e., non‐lesion) brain lesions across vigilance states.

No statistically significant differences emerged between the two groups after Wilcoxon rank‐sum testing with Benjamini‐Hochberg (BH) correction (all *p* > 0.05; Supplementry Figure 1). Therefore, patients with minor brain lesions were pooled with non‐lesional patients for the subsequent main analyses.

### 3.2 Vigilance States Distinctly Modulate Global Neuronal Oscillatory Dynamics

To examine the impact of vigilance states on neuronal oscillatory dynamics, we analysed power density, phase synchronisation, bistability, and cross‐frequency coupling across states.

**FIGURE 2:**
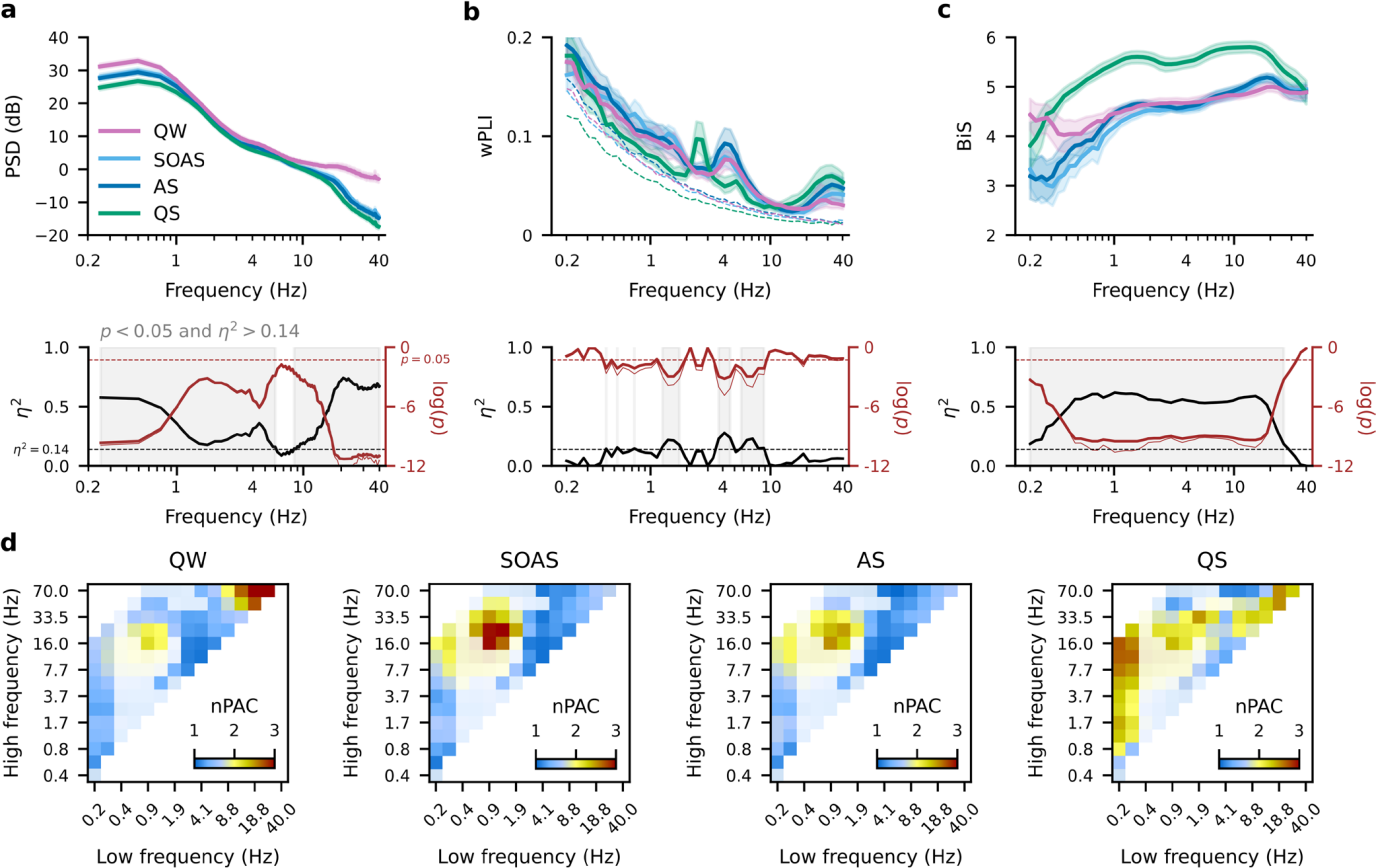
Neuronal dynamics are modulated by different vigilance states. (a) Power spectral density (PSD), (b) global weighted phase‐lag index (wPLI), and (c) global bistability index (BiS) across the different vigilance states. The shaded areas represent the variability computed from 100 bootstraps (95th percentile). The second row shows the spectra of effect size (η^2^, black) and *p*‐value (reddish) of Kruskal‐Wallis test for each frequency. The *p*‐value is represented as a thin line (uncorrected) and a thick line (BH‐corrected). Grey areas indicate η^2^ > 0.14 (large effect) and *p* < 0.05 after Benjamini‐Hochberg FDR correction. (d) Cohort‐averaged normalised phase‐amplitude coupling (nPAC) for each vigilance state. Warmer colours indicate stronger coupling, while cooler colours represent weaker coupling. nPAC matrices are alpha‐shaded after Kruskal‐Wallis test and Benjamini‐Hochberg FDR correction. Regions with less transparency correspond to areas with η^2^ > 0.14 (large effect) and BH‐corrected *p* < 0.05.

**FIGURE 3:**
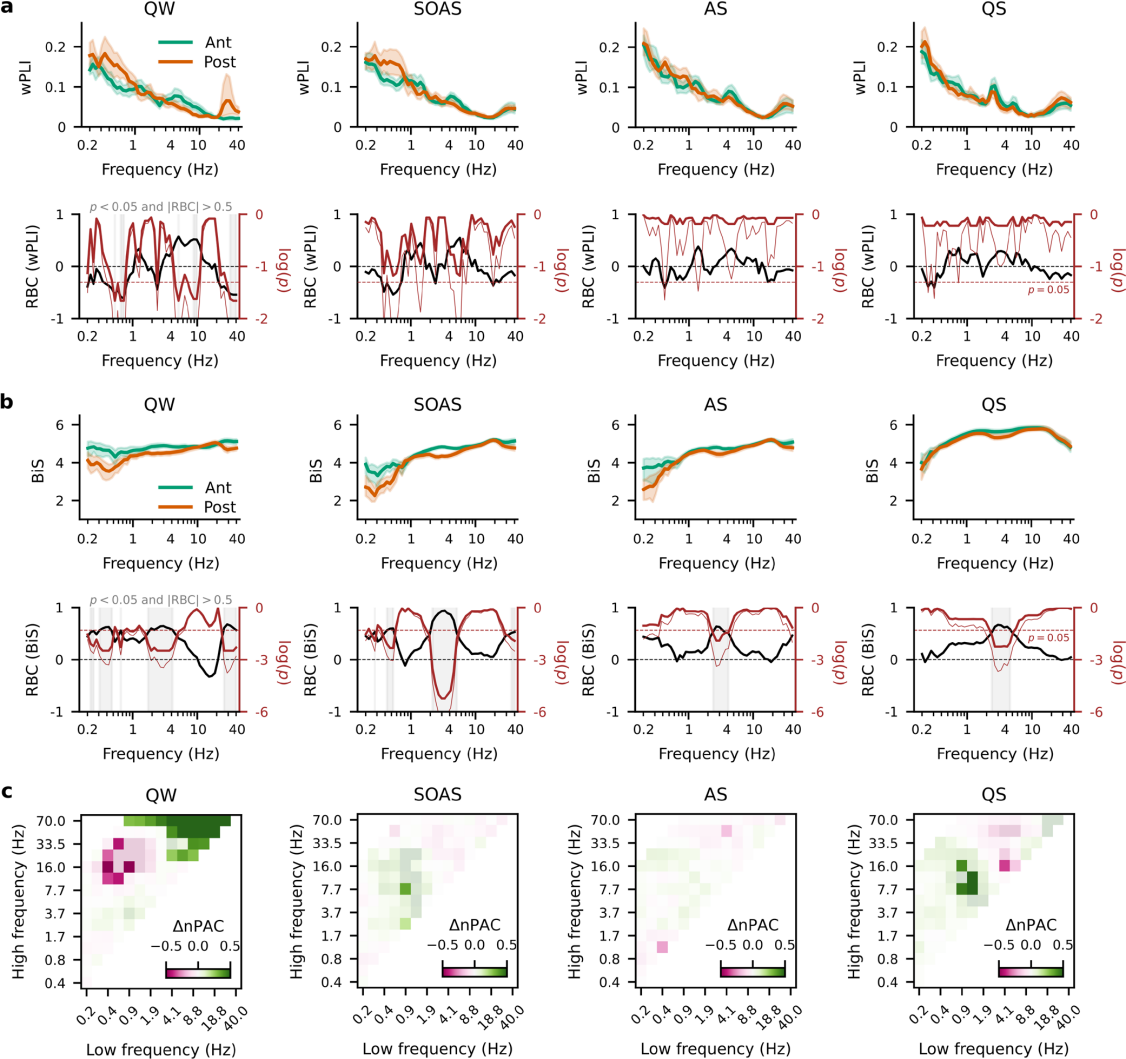
Neuronal dynamics differ between anterior and posterior derivations. (a) Comparison between anterior versus posterior weighted phase‐lag index (wPLI) and (b) bistability index (BiS), separately for each vigilance state. The shaded areas represent the variability computed from 100 bootstraps (95th percentile). The second row shows the spectra of effect size (rank‐biserial correlation coefficient – RBC, black) and *p*‐value (reddish) for each frequency. The *p*‐value is represented as a thin line (uncorrected) and a thick line (BH‐corrected). Grey areas indicate RBC > 0.5 (large effect) and *p* < 0.05 after Benjamini‐Hochberg FDR correction. (c) Difference between anterior and posterior normalised phase‐amplitude coupling (nPAC) for each vigilance state. Green hues indicate nPAC_Anterior_>nPAC_Posterior_, whereas pink hues indicate the opposite. Alpha shading was applied after pairwise Wilcoxon rank‐sum test and Benjamini‐Hochberg FDR correction. Regions with less transparency correspond to areas with RBC > 0.5 (large effect) and uncorrected *p* < 0.05.

Amplitude of local neuronal oscillations was affected by vigilance states, with QS showing the lowest power, followed by SOAS and AS, while QW exhibited the highest (*p* < 0.05, Kruskal‐Wallis test, BH FDR corrected) (Figure 2a; Pairwise differences shown in Supplementry Figure 2). These differences spanned the entire frequency range (0.2–40 Hz), except in the θ–α band (6–9 Hz), and with stronger effect size (η^2^ > 0.14) in the slow‐wave band (< 1 Hz) and β‐γ band (> 20 Hz). At a large‐scale level, neuronal oscillations seemed to interact in a state‐ and frequency‐specific manner at this early stage of life. Spectral analysis of global phase synchronisation revealed distinct wPLI peaks across different vigilance states (Figure 2b; Pairwise differences shown in Supplementry Figure 3). In QS, wPLI was significantly higher than in the other vigilance states in the δ band (2–3 Hz), while QW, SOAS, and AS showed higher wPLI in the θ band (4–6 Hz) (*p* < 0.05, Kruskal‐Wallis test, BH uncorrected).

**FIGURE 4:**
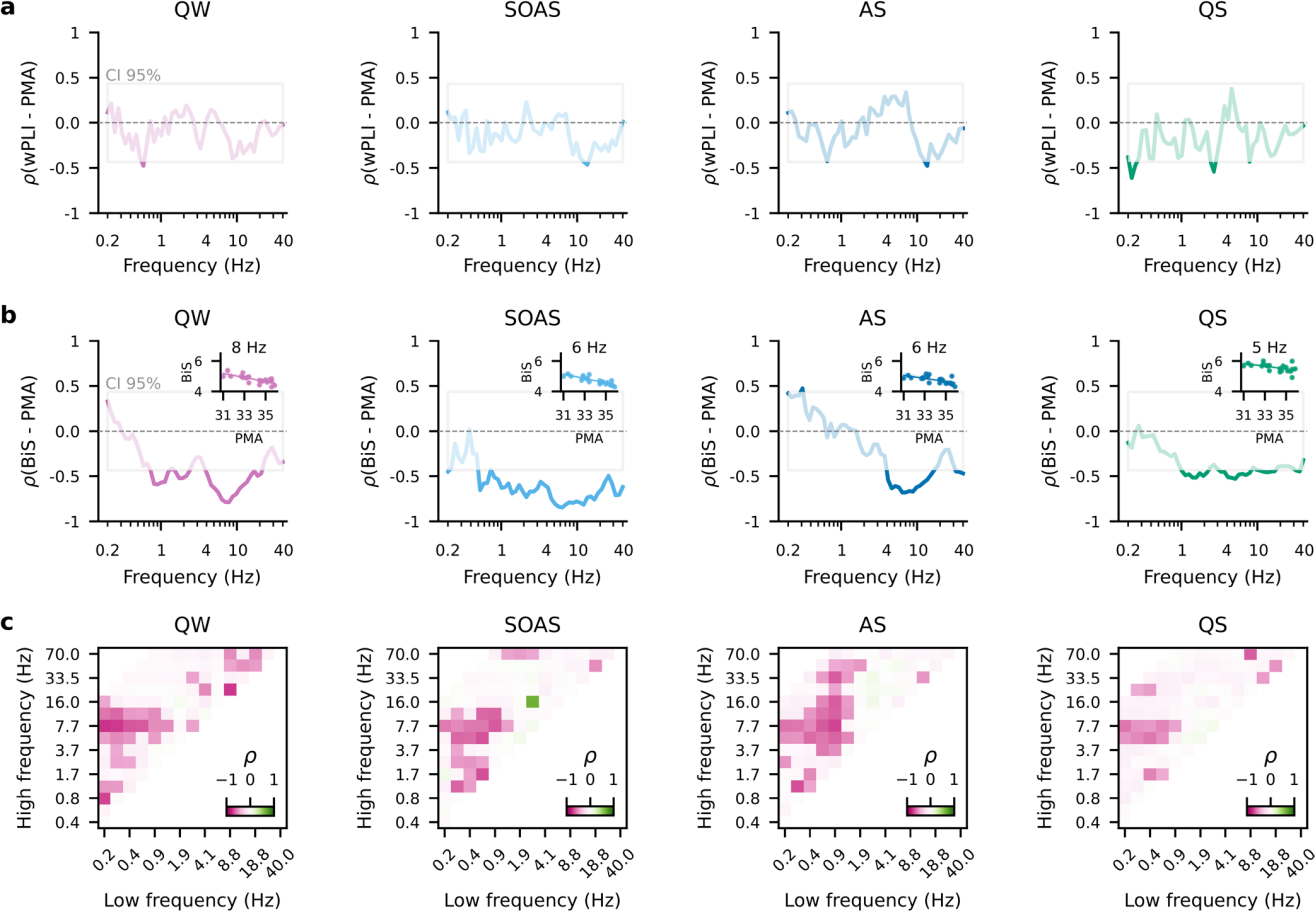
Neuronal dynamics correlate with age in specific vigilance states. (a) Spectra of Spearman correlation coefficient between weighted phase‐lag index (wPLI) and postmenstrual age (ρ(wPLI–PMA)) and (b) bistability index (BiS) and postmenstrual age (ρ(BiS–PMA)), separately for each vigilance state. Shaded areas represent the 2.5th and 97.5th percentiles of 10′000 surrogates. Negative correlations indicate that the metric decreases with age, as illustrated in each small panel in (b), where the minimum BiS‐PMA correlation was chosen as an example for each vigilance state. (c) Spearman correlation coefficient (ρ) between local normalised phase‐amplitude coupling (nPAC) and postmenstrual age for each low frequency–high frequency pair and vigilance state. Regions with less transparency correspond to areas with BH corrected *p* < 0.05.

Differences extended beyond phase synchronisation as we observed a significant difference in global bistability across vigilance states (*p* < 0.05, Kruskal‐Wallis test, BH corrected) (Figure 2c; Pairwise differences shown in Supplementry Figure 4). BiS values in QS were higher compared to other vigilance states across the frequency range of 0.3–30 Hz, with the strongest effect (η^2^ > 0.14) in the 0.5–12 Hz. SOAS and AS showed similarly lower BiS values in the slow‐oscillations band (< 0.5 Hz) compared to the other vigilance states. BiS values in QW in the low frequency range (< 1 Hz) were higher with respect to AS and SOAS.

PAC analysis also revealed frequency modulations (Figure 2d; Pairwise differences shown in Supplementry Figure 5). Quiet wakefulness displayed higher coupling between the phase of β oscillations (12–30 Hz) and γ amplitudes (55–70 Hz). In SOAS and AS, the phase of δ‐band oscillations (1–2 Hz) was coupled with β‐oscillation amplitudes (~16–25 Hz), with higher nPAC values in SOAS compared to AS. In contrast, QS showed a more widespread area of modulation between phases < 1 Hz and broadband oscillations (1–16 Hz) and between the phase of β oscillations (20–30 Hz) and γ amplitudes (55–70 Hz).

Altogether, these results revealed that: QW was characterised by the highest amplitudes in a wide range of frequencies, a broadband mild level of bistability, prominent peaks in θ‐band wPLI, and increased coupling between β and γ oscillations; SOAS and AS had similar characteristics with mid‐level amplitudes, a reduced bistability of slow oscillations, a peak in θ band phase synchronisation, and significant effect of slow‐wave oscillations in modulating β‐γ band amplitudes; finally, QS had the smallest amplitude, a significantly higher bistability across a broad range of frequencies, a prominent peak in wPLI at around 2 Hz, and significantly higher modulation between slow‐oscillation phases and the amplitudes of faster rhythms.

### 3.3 Neuronal Dynamics Reflect Spatial Differences in Posterior and Anterior Derivations

Next, we analysed whether neuronal dynamics differ between mid‐posterior (C3–O1, T3–O1, C4–O2, T4–O2, O1–O2) and mid‐anterior (Fp1–C3, Fp1–T3, Fp2–C4, Fp2–T4, Fp1–Fp2) derivations. We observed elevated wPLI levels in posterior derivations for frequencies < 1 Hz, during QW and SOAS (*p* < 0.05, Wilcoxon rank‐sum test, BH uncorrected) (Figure 3a). Posterior derivations exhibited lower levels of wPLI during QW and SOAS (*p* < 0.05, Wilcoxon rank‐sum test, BH uncorrected) in the 5–10 Hz range. Additionally, we observed a peak in wPLI in anterior derivations at 1 Hz during SOAS (*p* < 0.05, Wilcoxon rank‐sum test, BH uncorrected), absent in posterior derivations. No significant differences between posterior and anterior derivations were observed in QS.

Posterior derivations showed significantly lower values of BiS in the δ band (2–4 Hz) than anterior derivations across all the vigilance states (*p* < 0.05, Wilcoxon rank‐sum test, BH corrected) (Figure 3b). Moreover, quiet wakefulness was characterised by significantly lower BiS values in the posterior compared to anterior derivations in the low‐frequency range (< 1 Hz) and in β–γ bands (25–40 Hz) (*p* < 0.05, Wilcoxon rank‐sum test, BH corrected).

In QW, posterior derivations showed stronger δ‐to‐β/γ coupling, while anterior derivations exhibited stronger θ‐to‐β and γ coupling (*p* < 0.05, uncorrected). On the other hand, stronger anterior modulation emerged between δ and θ‐to‐α bands in QS. However, none of these PAC differences remained significant after correction for multiple comparisons (Figure 3c). To complement these regional comparisons, we repeated the analyses using a single frontal (Fp1–Fp2) and a single occipital (O1–O2) derivation (Supplementry Figure 6). In this configuration, we observed similar anterior–posterior differences for BiS values (Supplementry Figure 6b).

### 3.4 Bistability and Local Phase‐Amplitude Coupling Decrease With Age in Preterm Infants

We first examined the relationships of each metric (wPLI, BiS, and local nPAC) with PMA, vigilance state, and frequency as random factors separately (Table 2).

**TABLE 2:** Summary of the linear mixed‐effects model assessing the relationship between each dependent variable (wPLI, BiS, and nPAC), postmenstrual age (PMA), vigilance state, and sex.

(a) Dependent Variable: wPLI						
	Coefficient	Std. Err.	*z*	*P* > |z|	95% CI (L)	95% CI (U)
Intercept	0.288	0.349	0.824	0.410	−0.397	0.973
Stage[T.AS]*	0.162	0.064	2.543	0.011	0.037	0.288
Stage[T.QS]	−0.124	0.064	−1.943	0.052	−0.249	0.001
Stage[T.QW]	−0.026	0.064	−0.411	0.681	−0.151	0.099
Sex[T.M]	−0.023	0.048	−0.471	0.638	−0.117	0.072
PMA*	−0.045	0.023	−0.362	0.046	−0.090	−0.001
Group Var.	0.599	0.924				

(b) Dependent Variable: BiS						
	Coefficient	Std. Err.	*z*	*P* > |z|	95% CI (L)	95% CI (U)
Intercept*	−0.724	0.242	−2.988	0.003	−1.199	−0.249
Stage[T.AS]	0.113	0.105	1.077	0.281	−0.092	0.318
Stage[T.QS]*	1.134	0.105	10.830	< 0.001	0.928	1.339
Stage[T.QW]*	0.748	0.105	7.142	< 0.001	0.542	0.953
Sex[T.M]*	0.164	0.079	2.085	0.037	0.010	0.319
PMA*	−0.079	0.037	−2.112	0.035	−0.151	−0.006
Group Var.	0.262	0.252				

(c) Dependent Variable: local nPAC						
	Coefficient	Std. Err.	*z*	*P* > |z|	95% CI (L)	95% CI (U)
Intercept	−0.162	0.088	−1.845	0.065	−0.334	0.010
Stage[T.AS]	−0.088	0.060	−1.469	0.142	−0.206	0.029
Stage[T.QS]*	0.443	0.060	7.386	< 0.001	0.325	0.560
Stage[T.QW]*	−0.196	0.060	−3.276	0.001	−0.314	−0.079
Sex[T.M]	−0.020	0.045	−0.448	0.654	−0.109	0.068
PMA*	−0.168	0.021	−7.902	< 0.001	−0.210	−0.126
Group Var.	0.028	0.050				

*Note:* The table reports the estimated coefficients (Coefficient), standard errors (Std. Err.), z‐scores (z), *p*‐values (*P* > |z|), and 95% confidence intervals ([0.025, 0.975], (L) and (U), respectively) for each predictor. The intercept represents the expected value of the dependent variable for the reference category (SOAS for vigilance states; female for sex). The coefficients for ‘Stage’ indicate the differences in the dependent variable relative to SOAS, while ‘Sex [T.M]’ represents the difference between males and females. ‘Group Var.’ denotes the variance of the random effect (frequency bands). Statistically significant results (*p* < 0.05) are marked with an asterisk (*).

For wPLI, PMA showed a small but significant negative association (β = −0.045, *p* = 0.046), suggesting a slight decrease in phase synchronisation with increasing age, while vigilance state and sex had no significant effects, except for AS showing higher wPLI compared to SOAS (β = 0.162, *p* = 0.011) (Table 2a). For BiS, we observed significant differences across vigilance states: QS and QW exhibited higher bistability compared to SOAS, whereas AS did not differ significantly. Male infants showed slightly higher BiS than females, and PMA was negatively associated with BiS (β = −0.079, *p* = 0.035), indicating a decrease in bistability with cortical maturation (Table 2b).

Similarly, local nPAC was strongly negatively associated with PMA (β = −0.168, *p* < 0.001), while no significant differences were found between male and female infants (Table 2c).

We observed a broadband effect of such negative correlations for both BiS and local nPAC (Figure 4). Maximal negative correlation for BiS occurs in θ band in all the vigilance states (Figure 4b). For local PAC, correlation with PMA was more evident between the phase of slow oscillations up to the δ‐band (0.2–2 Hz) and the amplitude of faster oscillations up to β across all vigilance states, with QS being the less correlated (Figure 4c).

## 4 Discussion

In this study, we asked whether electrophysiological markers of cortical activity recorded using low‐density EEG exhibit distinct spatial and temporal patterns that might reflect early cortical maturation in preterm infants. We found that these markers follow distinct developmental trajectories. Vigilance states influence network dynamics: quiet sleep (QS) shows higher bistability and δ‐band phase synchronisation, whereas quiet wakefulness (QW) is associated with β‐to‐γ coupling. Anterior regions show higher bistability than posterior regions, consistent with known patterns of delayed frontal maturation.

Also, bistability and phase‐amplitude coupling decrease with increasing postmenstrual age (PMA), indicating a transition from discontinuous, burst‐driven activity toward more continuous oscillatory patterns typical of a maturing cortex. These results advance the construct of cortical maturation by providing objective, state‐specific EEG metrics that track developmental progression even using a low‐density EEG montage, representing a readily applicable application in clinical monitoring. Our findings offer a reference framework for future research and may help identify potential biomarkers to detect atypical trajectories and support early intervention strategies in preterm populations.

### 4.1 Vigilance States Modulate Cortical Dynamics in Preterm Infants

Quiet wakefulness (QW) exhibited elevated broadband power, particularly in the β–low γ range (Figure 2a), and increased bistability (Figure 2c). While these features could reflect heightened sensorimotor processing and immature state differentiation (Norman et al. 2008; Saby and Marshall 2012; Wang et al. 2023), the presence of slow oscillations (< 1 Hz) during QW (Figure 2a), combined with the rarity of this state at this age (Uccella et al. 2025), suggests that QW may represent transitional or drowsy states under elevated sleep pressure rather than fully alert wakefulness.

By contrast, QS emerged as a distinct and highly organised brain state characterised by low power, high bistability, and strong δ‐band synchronisation. This pattern is consistent with previous descriptions of discontinuous thalamocortical activity (González et al. 2011; Tokariev et al. 2012) and large‐scale slow oscillations during sleep (Vanhatalo and Kaila 2006; Yrjölä et al. 2022). Together, these features portray QS as a state in which transient network disconnection alternates with synchronised bursts, an early cortical activity pattern implicated in shaping synaptic connectivity and supporting developmental plasticity (Steriade et al. 1993; McCormick and Bal 1997; Khazipov and Luhmann 2006; Colonnese and Khazipov 2012). The overall reduction in spectral power may thus reflect this discontinuous structure, despite the presence of intermittent slow‐wave bursts indicated by higher BiS.

Moreover, our findings further highlight the structured nature of QS, with slow rhythms organising the timing of higher‐frequency activity. The presence of δ‐to‐higher‐frequency PAC reinforces the interpretation of QS as a key window for network reorganisation during early development (Varley et al. 2025).

Active sleep (AS) and sleep onset active sleep (SOAS) showed reduced bistability compared to QS and pronounced θ‐band synchronisation (Figure 2). This pattern resembles the spectral signature of adult REM sleep (Moroni et al. 2007; Boyce et al. 2016) and may represent early developmental precursors of REM (Roffwarg et al. 1966; de Groot et al. 2024), consistent with a trajectory of maturation in state‐dependent network dynamics.

These observations are consistent with previous studies (Yrjölä et al. 2022; Yrjölä et al. 2024) regarding vigilance‐state‐specific frequency patterns, with δ‐band synchronisation peaks during QS and θ‐band peaks during AS.

In addition to global and regional phase‐amplitude coupling (PAC) trends, our analysis of coupling patterns revealed important state‐dependent differences in the hierarchical organisation of cortical coordination. During QS, PAC was predominantly driven by slow oscillations modulating the amplitude of higher‐frequency activities (δ‐to‐β/γ), underscoring the orchestrating role of slow rhythms during early brain development (Dreyfus‐Brisac and Monod 1965; Curzi‐Dascalova 1992; Vanhatalo et al. 2005; Tolonen et al. 2007). In contrast, QW was characterised by enhanced fast‐to‐fast coupling (e.g., β phase modulating γ amplitude), suggesting that local cortical circuits were more actively engaged and regulated in this state. Both AS and SOAS exhibited an intermediate profile, with both slow‐to‐fast and fast‐to‐fast PAC present but at lower levels. These findings highlight a dynamic reorganisation of cortical coupling across vigilance states, reflecting developmental and functional shifts from global network coordination during sleep to more local, fast‐frequency interactions during wakefulness.

### 4.2 Regional Differences in Bistability and PAC

Beyond state‐dependent modulation, regional differences also emerged. Anterior derivations exhibited higher bistability of δ oscillations across all states, in line with the posterior‐to‐anterior gradient of cortical maturation (Ruoss et al. 2001; Ramenghi et al. 2007; Kurth et al. 2012; van den Heuvel and Thomason 2016). This pattern likely reflects the persistence of immature, burst‐like activity in prefrontal areas. Furthermore, the anterior predominance of δ‐band bistability likely mirrors the role of delta brushes and slow oscillations in orchestrating frontal network development (Arichi et al. 2017; Whitehead et al. 2017).

PAC profiles revealed regionally heterogeneous trajectories (Figure 3c). Although statistical significance was not reached for anterior–posterior PAC differences following FDR correction, our analyses indicated stronger slow‐to‐fast coupling in posterior regions during QW. On the other hand, PAC from anterior regions showed higher β‐to‐γ PAC in QW and state‐dependent slow waves to *θ*/*σ* range modulation.

### 4.3 Maturational Trajectories and Developmental Shifts in Criticality

A central finding of our study is that bistability and PAC declined systematically with advancing PMA. This trajectory likely reflects a gradual reorganisation of cortical network dynamics, through which initially rigid and globally synchronised network states are progressively replaced by more independent and adaptable patterns of oscillatory coordination. This interpretation is supported by recent studies spanning different stages of early brain development (Yrjölä et al. 2024). In very premature infants (24–27 weeks gestational age), emerging phase‐amplitude coupling has been observed between slow waves and nested theta oscillations, reflecting an endogenous, sensory‐independent mechanism thought to support the initial wiring of perisylvian networks (Moghimi et al. 2020). Later in development, global neuronal coupling modes, including amplitude‐amplitude correlations and distant PAC, have been shown to decline with increasing conceptional age, indicating reduced globally coherent bursting and increasing local autonomy of cortical networks (Yrjölä et al. 2024).

Accordingly, our data indicate that, in our cohort of preterm infants, local PAC remains prominent but shows a systematic decline with advancing PMA, alongside dynamic modulation by vigilance states and regional differentiation. This pattern reflects a transitional stage between globally coupled, burst‐dominated dynamics and the emergence of more autonomous and flexible oscillatory regimes. However, as highlighted by the observational and correlational nature of our and previous studies (Moghimi et al. 2020), mechanistic conclusions remain inherently limited. Establishing causal relationships between these coupling modes and cortical maturation will require complementary approaches, including experimental perturbations and multimodal neuroimaging strategies.

Our findings align with and significantly extend the brain criticality hypothesis, which posits that healthy neural systems operate near a critical regime, balancing order and disorder (Zimmern 2020; Hengen and Shew 2025). The gradual decline in bistability and PAC that we observed with advancing PMA likely reflects a developmental tuning process that progressively guides cortical networks toward this critical regime. Early in development, the preterm brain appears to operate in a relatively supercritical state, characterised by hypersynchronisation, elevated burst propensity, and strong hierarchical coupling (Hartley et al. 2012; Iyer et al. 2015). Such dynamics, while seemingly inefficient from the perspective of mature network function, may be essential during early life to drive activity‐dependent wiring and synaptic refinement across widespread circuits.

As maturation progresses, the observed decline in PAC (particularly slow‐to‐fast coupling) and reduced bistability suggest a shift toward more distributed and autonomous oscillatory regimes. This transition may serve to prevent excessive excitation and allow the brain to achieve a dynamic balance where oscillations can flexibly couple and decouple in response to environmental demands. In this sense, our data suggest that the developing brain undergoes a form of “critical tuning”, through which early hypersynchronous states evolve into more context‐sensitive and efficient modes of operation. Importantly, the state‐dependent and regionally differentiated patterns observed in our cohort highlight that criticality is not a global property achieved uniformly across the cortex, but rather a multifaceted and spatially heterogeneous process.

Taken together, these findings position criticality not as a static target of brain maturation, but as a dynamic developmental trajectory involving progressive shifts in local and global network properties. Disruption or deviation from this trajectory, either through premature transition to hypoconnected states or failure to downregulate hypersynchronous dynamics, may have important implications for neurodevelopmental outcomes in preterm populations. Future studies integrating multimodal imaging, computational modelling, and longitudinal clinical follow‐up will be crucial to further elucidate how critical tuning processes support or impair emerging cognitive, sensory, and motor functions.

### 4.4 Study Limitations

This study included 21 preterm infants, a relatively small cohort that reflects the inherent challenges of recruiting and recording EEG in this vulnerable population. While the sample size may limit generalizability and robustness of group‐level comparisons, a post hoc power assessment indicated that, for the observed correlation values, the study achieved a power exceeding 90%. Moreover, the availability of 24‐h PSG recordings allowed us to extract well‐defined segments from multiple vigilance states, including quiet wakefulness—an advantage over previous studies based on shorter nap recordings, which typically assessed only active and quiet sleep.

High‐density EEG caps (≥ 32 channels) would have provided higher spatial resolution, allowing a better characterisation of large‐scale network organisation during early brain development. However, we chose an 8‐channel setup to ensure stable recordings in a clinically feasible configuration—well‐suited for fragile preterm infants and compatible with long‐term monitoring in the neonatal intensive care setting.

We acknowledge that sensor‐level analysis inherently reduces spatial specificity; however, all our analyses were performed at the sensor level, as the limited number of EEG channels and the lack of individual MRI data precluded reliable source reconstruction.

## 5 Conclusion

In summary, our results provide a comprehensive account of how vigilance states, cortical regions, and age interact to shape early cortical dynamics in preterm infants. Decreasing bistability and PAC reflect the gradual emergence of autonomous cortical activity, while regional differences align with known maturation gradients. Moreover, state‐dependent reorganisation of PAC patterns, from slow‐dominated during sleep to fast‐to‐fast coupling during wakefulness, highlights the functional adaptation of oscillatory coordination during early life. Future research with larger samples and high‐density recordings is needed to validate and expand on these findings. In a longitudinal study, these quantitative EEG metrics may prove useful for describing aberrant brain maturation.

## Author Contributions

**Monica Roascio:** writing – review and editing, methodology. **Sara Uccella:** conceptualization, investigation, writing – original draft, writing – review and editing, data curation, resources, supervision, methodology, funding acquisition, project administration.

## Funding

This work was supported by NEXTGENERATIONEU and funded by Ministero dell'Università e della Ricerca, National Recovery and Resilience plan, project MNESYS ‐ a Multiscale integrated approach to the study of the nervous system in health and diseases (PE0000006); University Research Funds – Genoa (SWAN project); 5 × 1000 IRCCS Gaslini.

## Conflicts of Interest

The authors declare no conflicts of interest.

## Supporting information


**Data S1:** jsr70308‐sup‐0001‐supinfo.docx. **Supporting Information**.
Supplementary Figure 1: No group differences in neuronal dynamics are observed between preterm infants with and without brain lesions. Comparison between non‐lesional and lesional infants of (a) weighted phase‐lag index (wPLI) and (b) bistability index (BiS), shown separately for each vigilance state. Shaded areas represent variability estimated from 100 bootstraps (95th percentile). The second row reports the spectra of effect size (rank‐biserial correlation coefficient, RBC; black) and Benjamini‐Hochberg‐corrected *p*‐values (red), with the thin dashed line indicating *p* = 0.05. (c) RBC values for normalised phase–amplitude coupling (nPAC) between non‐lesional and lesional infants for each vigilance state. Positive RBC values indicate higher values in non‐lesional compared to lesional infants (green), while negative values indicate the opposite pattern (pink). More transparent regions correspond to non‐significant differences (*p* > 0.05, Benjamini‐Hochberg corrected).
Supplementary Figure 2: Pairwise Wilcoxon rank‐sum test comparing the PSD across sleep stages. Empty circles indicate *p* < 0.05 after the Wilcoxon rank‐sum test, while filled circles represent *p* < 0.05 after Benjamini‐Hochberg (BH) correction. Legend. PSD: power spectral density; QW: quiet wakefulness; SOAS: sleep onset active sleep; AS: active sleep; QS: quiet sleep.
Supplementary Figure 3: Pairwise Wilcoxon rank‐sum test comparing the wPLI across sleep stages. Empty circles indicate *p* < 0.05 after the Wilcoxon rank‐sum test, while filled circles represent *p* < 0.05 after Benjamini‐Hochberg (BH) correction. Legend. wPLI: weighted phase‐lag index; QW: quiet wakefulness; SOAS: sleep onset active sleep; AS: active sleep; QS: quiet sleep.
Supplementary Figure 4: Pairwise Wilcoxon rank‐sum test comparing the BiS across sleep stages. Empty circles indicate *p* < 0.05 after the Wilcoxon rank‐sum test, while filled circles represent *p* < 0.05 after Benjamini‐Hochberg (BH) correction. Legend. BiS: bistability index; QW: quiet wakefulness; SOAS: sleep onset active sleep; AS: active sleep; QS: quiet sleep.
Supplementary Figure 5: Difference between each pair of vigilance states nPAC. Alpha shading was applied after pairwise Wilcoxon rank‐sum test and Benjamini‐Hochberg FDR correction. Regions with less transparency correspond to areas with uncorrected *p* < 0.05. Legend. nPAC: normalised phase‐amplitude coupling; QW: quiet wakefulness; SOAS: sleep onset active sleep; AS: active sleep; QS: quiet sleep.
Supplementary Figure 6: Neuronal dynamics differ from occipital (O1‐O2) to frontal (Fp1‐Fp2) derivations. (a) Comparison between frontal and occipital eigenvector centrality (EVC) and (b) BiS, separately for each vigilance state. The shaded areas represent the variability computed from 100 bootstraps (95th percentile). The second row shows the spectra of effect size (Cohen's d, black) and *p*‐value (reddish) for each frequency. The *p*‐value is represented as a thin line (uncorrected) and a thick line (BH‐corrected). Grey areas indicate d > 0.8 (large effect) and *p* < 0.05 after Benjamini‐Hochberg FDR correction. (c) Difference between anterior and posterior nPAC for each vigilance state. Green hues indicate nPAC_Anterior_>nPAC_Posterior_, whereas pink hues indicate the opposite. Alpha shading was applied after pairwise Wilcoxon rank‐sum test and Benjamini‐Hochberg FDR correction. Regions with less transparency correspond to areas with d > 0.8 (large effect) and uncorrected *p* < 0.05. Legend. EVC: eigenvector centrality; BiS: bistability index; nPAC: normalised phase‐amplitude coupling; QW: quiet wakefulness; SOAS: sleep onset active sleep; AS: active sleep; QS: quiet sleep.

## Data Availability

The data used in this work can be made available upon a reasonable request due to privacy issues of clinical data.

## References

[jsr70308-bib-0001] André, M. , M.‐D. Lamblin , A. M. d'Allest , et al. 2010. “Electroencephalography in Premature and Full‐Term Infants. Developmental Features and Glossary.” Clinical Neurophysiology 40: 59–124. 20510792 10.1016/j.neucli.2010.02.002

[jsr70308-bib-0002] Arichi, T. , K. Whitehead , G. Barone , et al. 2017. “Localization of Spontaneous Bursting Neuronal Activity in the Preterm Human Brain With Simultaneous EEG‐fMRI.” eLife 6: e27814. 28893378 10.7554/eLife.27814 PMC5595428

[jsr70308-bib-0003] Bertino, E. , E. Spada , L. Occhi , et al. 2010. “Neonatal Anthropometric Charts: The Italian Neonatal Study Compared With Other European Studies.” Journal of Pediatric Gastroenterology and Nutrition 51: 353–361. 20601901 10.1097/MPG.0b013e3181da213e

[jsr70308-bib-0004] Blumberg, M. S. , J. C. Dooley , and A. Tiriac . 2022. “Sleep, Plasticity, and Sensory Neurodevelopment.” Neuron 110: 3230–3242. 36084653 10.1016/j.neuron.2022.08.005 PMC9588561

[jsr70308-bib-0005] Blumberg, M. S. , J. A. Lesku , P.‐A. Libourel , M. H. Schmidt , and N. C. Rattenborg . 2020. “What Is REM Sleep?” Current Biology 30: R38–R49. 31910377 10.1016/j.cub.2019.11.045 PMC6986372

[jsr70308-bib-0006] Boyce, R. , S. D. Glasgow , S. Williams , and A. Adamantidis . 2016. “Causal Evidence for the Role of REM Sleep Theta Rhythm in Contextual Memory Consolidation.” Science 352: 812–816. 27174984 10.1126/science.aad5252

[jsr70308-bib-0007] Burlando, G. , C. Belforte , F. Siebenhühner , et al. 2025. “Sleep‐Modulated Cross‐Frequency Coupling Between δ Phase and β–γ Bistability: A System‐Level Modulation of Epileptic Activity.” bioRxiv. 10.1101/2025.03.25.642592v1.

[jsr70308-bib-0008] Buzsáki, G. , and A. Draguhn . 2004. “Neuronal Oscillations in Cortical Networks.” Science 304: 1926–1929. 15218136 10.1126/science.1099745

[jsr70308-bib-0009] Canolty, R. T. , and R. T. Knight . 2010. “The Functional Role of Cross‐Frequency Coupling.” Trends in Cognitive Sciences 14: 506–515. 20932795 10.1016/j.tics.2010.09.001 PMC3359652

[jsr70308-bib-0010] Chialvo, D. R. 2010. “Emergent Complex Neural Dynamics.” Nature Physics 6: 744–750.

[jsr70308-bib-0011] Cocchi, L. , L. L. Gollo , A. Zalesky , and M. Breakspear . 2017. “Criticality in the Brain: A Synthesis of Neurobiology, Models and Cognition.” Progress in Neurobiology 158: 132–152. 28734836 10.1016/j.pneurobio.2017.07.002

[jsr70308-bib-0012] Cohen, B. H. 2008. Explaining Psychological Statistics. John Wiley & Sons.

[jsr70308-bib-0013] Cohen, J. 2013. Statistical Power Analysis for the Behavioral Sciences. Routledge.

[jsr70308-bib-0014] Colonnese, M. , and R. Khazipov . 2012. “Spontaneous Activity in Developing Sensory Circuits: Implications for Resting State fMRI.” NeuroImage 62: 2212–2221. 22387472 10.1016/j.neuroimage.2012.02.046

[jsr70308-bib-0015] Curzi‐Dascalova, L. 1992. “Physiological Correlates of Sleep Development in Premature and Full‐Term Neonates.” Clinical Neurophysiology 22: 151–166. 1630415 10.1016/s0987-7053(05)80751-8

[jsr70308-bib-0016] de Groot, E. R. , X. Wang , K. Wojtal , et al. 2024. “Association Between Sleep Stages and Brain Microstructure in Preterm Infants: Insights From DTI Analysis.” Sleep Medicine 121: 336–342. 39053129 10.1016/j.sleep.2024.07.021

[jsr70308-bib-0017] Dreyfus‐Brisac, C. , and N. Monod . 1965. “Sleep of Premature and Full‐Term Neonates—A POLYGRAPHIC Study.” Proceedings of the Royal Society of Medicine 58: 6–7. 14269756 10.1177/003591576505800104 PMC1898300

[jsr70308-bib-0018] Dubois, J. , G. Dehaene‐Lambertz , S. Kulikova , C. Poupon , P. S. Hüppi , and L. Hertz‐Pannier . 2014. “The Early Development of Brain White Matter: A Review of Imaging Studies in Fetuses, Newborns and Infants.” Neuroscience 276: 48–71. 24378955 10.1016/j.neuroscience.2013.12.044

[jsr70308-bib-0019] Frank, M. G. 2017. “Sleep and Plasticity in the Visual Cortex: More Than Meets the Eye.” Current Opinion in Neurobiology 44: 8–12. 28126451 10.1016/j.conb.2017.01.001 PMC7699671

[jsr70308-bib-0020] Freyer, F. , K. Aquino , P. A. Robinson , P. Ritter , and M. Breakspear . 2009. “Bistability and Non‐Gaussian Fluctuations in Spontaneous Cortical Activity.” Journal of Neuroscience 29: 8512–8524. 19571142 10.1523/JNEUROSCI.0754-09.2009 PMC6665653

[jsr70308-bib-0021] Freyer, F. , J. A. Roberts , R. Becker , P. A. Robinson , P. Ritter , and M. Breakspear . 2011. “Biophysical Mechanisms of Multistability in Resting‐State Cortical Rhythms.” Journal of Neuroscience 31: 6353–6361. 21525275 10.1523/JNEUROSCI.6693-10.2011 PMC6622680

[jsr70308-bib-0022] Fries, P. 2005. “A Mechanism for Cognitive Dynamics: Neuronal Communication Through Neuronal Coherence.” Trends in Cognitive Sciences 9: 474–480. 16150631 10.1016/j.tics.2005.08.011

[jsr70308-bib-0023] González, J. J. , S. Mañas , L. De Vera , et al. 2011. “Assessment of Electroencephalographic Functional Connectivity in Term and Preterm Neonates.” Clinical Neurophysiology 122: 696–702. 21074493 10.1016/j.clinph.2010.08.025

[jsr70308-bib-0024] Grigg‐Damberger, M. M. 2016. “The Visual Scoring of Sleep in Infants 0 to 2 Months of Age.” Journal of Clinical Sleep Medicine 12: 429–445. 26951412 10.5664/jcsm.5600 PMC4773630

[jsr70308-bib-0025] Haldeman, C. , and J. M. Beggs . 2005. “Critical Branching Captures Activity in Living Neural Networks and Maximizes the Number of Metastable States.” Physical Review Letters 94: 058101. 15783702 10.1103/PhysRevLett.94.058101

[jsr70308-bib-0026] Hartley, C. , L. Berthouze , S. R. Mathieson , et al. 2012. “Long‐Range Temporal Correlations in the EEG Bursts of Human Preterm Babies.” PLoS One 7: e31543. 22363669 10.1371/journal.pone.0031543 PMC3283672

[jsr70308-bib-0027] Hengen, K. B. , and W. L. Shew . 2025. “Is Criticality a Unified Setpoint of Brain Function?” Neuron 113: 2582–2598.e2. 40555236 10.1016/j.neuron.2025.05.020 PMC12374783

[jsr70308-bib-0028] Iyer, K. K. , J. A. Roberts , L. Hellström‐Westas , et al. 2015. “Cortical Burst Dynamics Predict Clinical Outcome Early in Extremely Preterm Infants.” Brain: A Journal of Neurology 138: 2206–2218. 26001723 10.1093/brain/awv129

[jsr70308-bib-0029] Jakab, A. , E. Schwartz , G. Kasprian , et al. 2014. “Fetal Functional Imaging Portrays Heterogeneous Development of Emerging Human Brain Networks.” Frontiers in Human Neuroscience 8: 852. 25374531 10.3389/fnhum.2014.00852 PMC4205819

[jsr70308-bib-0030] Kerby, D. S. 2014. “The Simple Difference Formula: An Approach to Teaching Nonparametric Correlation1.” Comprehensive Psychology 3: 11.IT.3.1.

[jsr70308-bib-0031] Khazipov, R. , and H. J. Luhmann . 2006. “Early Patterns of Electrical Activity in the Developing Cerebral Cortex of Humans and Rodents.” Trends in Neurosciences 29: 414–418. 16713634 10.1016/j.tins.2006.05.007

[jsr70308-bib-0032] Kinouchi, O. , and M. Copelli . 2006. “Optimal Dynamical Range of Excitable Networks at Criticality.” Nature Physics 2: 348–351.

[jsr70308-bib-0033] Kurth, S. , M. Ringli , M. K. Lebourgeois , et al. 2012. “Mapping the Electrophysiological Marker of Sleep Depth Reveals Skill Maturation in Children and Adolescents.” NeuroImage 63: 959–965. 22498654 10.1016/j.neuroimage.2012.03.053 PMC4444061

[jsr70308-bib-0034] Lotfi, N. , T. Feliciano , L. A. A. Aguiar , et al. 2021. “Statistical Complexity Is Maximized Close to Criticality in Cortical Dynamics.” Physical Review E 103: 012415. 33601583 10.1103/PhysRevE.103.012415

[jsr70308-bib-0035] Malova, M. , A. Parodi , M. Severino , et al. 2024. “Neurodevelopmental Outcome at 3 Years of Age in Very Low Birth Weight Infants According to Brain Development and Lesions.” Current Pediatric Reviews 20: 94–105. 36752291 10.2174/1573396319666230208092416

[jsr70308-bib-0036] Marlow, N. , Y. Ni , R. Lancaster , et al. 2021. “No Change in Neurodevelopment at 11 Years After Extremely Preterm Birth.” Archives of Disease in Childhood. Fetal and Neonatal Edition 106: 418–424. 33504573 10.1136/archdischild-2020-320650

[jsr70308-bib-0037] McCormick, D. A. , and T. Bal . 1997. “Sleep and Arousal: Thalamocortical Mechanisms.” Annual Review of Neuroscience 20: 185–215. 10.1146/annurev.neuro.20.1.185 9056712

[jsr70308-bib-0038] McQuillen, P. S. , and D. M. Ferriero . 2005. “Perinatal Subplate Neuron Injury: Implications for Cortical Development and Plasticity.” Brain Pathology 15: 250–260. 16196392 10.1111/j.1750-3639.2005.tb00528.x PMC8096042

[jsr70308-bib-0039] Meisel, C. , A. Storch , S. Hallmeyer‐Elgner , E. Bullmore , and T. Gross . 2012. “Failure of Adaptive Self‐Organized Criticality During Epileptic Seizure Attacks.” PLoS Computational Biology 8: e1002312. 22241971 10.1371/journal.pcbi.1002312 PMC3252275

[jsr70308-bib-0040] Moghimi, S. , A. Shadkam , M. Mahmoudzadeh , et al. 2020. “The Intimate Relationship Between Coalescent Generators in Very Premature Human Newborn Brains: Quantifying the Coupling of Nested Endogenous Oscillations.” Human Brain Mapping 41: 4691–4703. 33463873 10.1002/hbm.25150 PMC7555093

[jsr70308-bib-0041] Moroni, F. , L. Nobili , G. Curcio , et al. 2007. “Sleep in the Human Hippocampus: A Stereo‐EEG Study.” PLoS One 2: e867. 17848998 10.1371/journal.pone.0000867 PMC1959185

[jsr70308-bib-0042] Norman, E. , I. Rosén , S. Vanhatalo , et al. 2008. “Electroencephalographic Response to Procedural Pain in Healthy Term Newborn Infants.” Pediatric Research 64: 429–434. 18594483 10.1203/PDR.0b013e3181825487

[jsr70308-bib-0043] Palva, J. M. , and S. Palva . 2018. “Functional Integration Across Oscillation Frequencies by Cross‐Frequency Phase Synchronization.” European Journal of Neuroscience 48: 2399–2406. 29094462 10.1111/ejn.13767

[jsr70308-bib-0044] Palva, J. M. , S. Palva , and K. Kaila . 2005. “Phase Synchrony Among Neuronal Oscillations in the Human Cortex.” Journal of Neuroscience 25: 3962–3972. 15829648 10.1523/JNEUROSCI.4250-04.2005 PMC6724920

[jsr70308-bib-0045] Palva, J. M. , S. H. Wang , S. Palva , et al. 2018. “Ghost Interactions in MEG/EEG Source Space: A Note of Caution on Inter‐Areal Coupling Measures.” NeuroImage 173: 632–643. 29477441 10.1016/j.neuroimage.2018.02.032

[jsr70308-bib-0046] Perin, J. , A. Mulick , D. Yeung , et al. 2022. “Global, Regional, and National Causes of Under‐5 Mortality in 2000–19: An Updated Systematic Analysis With Implications for the Sustainable Development Goals.” Lancet Child & Adolescent Health 6: 106–115. 34800370 10.1016/S2352-4642(21)00311-4 PMC8786667

[jsr70308-bib-0047] Ramenghi, L. A. , M. Fumagalli , A. Righini , et al. 2007. “Magnetic Resonance Imaging Assessment of Brain Maturation in Preterm Neonates With Punctate White Matter Lesions.” Neuroradiology 49: 161–167. 17119946 10.1007/s00234-006-0176-y

[jsr70308-bib-0048] Raudenbush, S. W. , and A. S. Bryk . 2002. Hierarchical Linear Models: Applications and Data Analysis Methods. SAGE.

[jsr70308-bib-0049] Roffwarg, H. P. , J. N. Muzio , and W. C. Dement . 1966. “Ontogenetic Development of the Human Sleep‐Dream Cycle.” Science 152: 604–619. 17779492 10.1126/science.152.3722.604

[jsr70308-bib-0050] Ruoss, K. , K. Lövblad , G. Schroth , A. C. Moessinger , and C. Fusch . 2001. “Brain Development (Sulci and Gyri) as Assessed by Early Postnatal MR Imaging in Preterm and Term Newborn Infants.” Neuropediatrics 32: 69–74. 11414646 10.1055/s-2001-13871

[jsr70308-bib-0051] Saby, J. N. , and P. J. Marshall . 2012. “The Utility of EEG Band Power Analysis in the Study of Infancy and Early Childhood.” Developmental Neuropsychology 37: 253–273. 22545661 10.1080/87565641.2011.614663 PMC3347767

[jsr70308-bib-0052] Shiraki, A. , H. Kidokoro , H. Watanabe , et al. 2024. “Sleep State‐Dependent Development of Resting‐State Functional Connectivity During the Preterm Period.” Sleep 47: zsae225. 39320057 10.1093/sleep/zsae225 PMC11632190

[jsr70308-bib-0053] Siebenhühner, F. , S. H. Wang , G. Arnulfo , et al. 2020. “Genuine Cross‐Frequency Coupling Networks in Human Resting‐State Electrophysiological Recordings.” PLoS Biology 18: e3000685. 32374723 10.1371/journal.pbio.3000685 PMC7233600

[jsr70308-bib-0054] Steriade, M. , D. A. McCormick , and T. J. Sejnowski . 1993. “Thalamocortical Oscillations in the Sleeping and Aroused Brain.” Science 262: 679–685. 8235588 10.1126/science.8235588

[jsr70308-bib-0055] Stevenson, N. J. , K. Tapani , L. Lauronen , and S. Vanhatalo . 2019. “A Dataset of Neonatal EEG Recordings With Seizure Annotations.” Scientific Data 6: 190039. 30835259 10.1038/sdata.2019.39 PMC6400100

[jsr70308-bib-0056] Tallon‐Baudry, C. , O. Bertrand , C. Delpuech , and J. Pernier . 1996. “Stimulus Specificity of Phase‐Locked and Non‐Phase‐Locked 40 Hz Visual Responses in Human.” Journal of Neuroscience 16: 4240–4249. 8753885 10.1523/JNEUROSCI.16-13-04240.1996 PMC6579008

[jsr70308-bib-0057] Thomason, M. E. , J. A. Brown , M. T. Dassanayake , et al. 2014. “Intrinsic Functional Brain Architecture Derived From Graph Theoretical Analysis in the Human Fetus.” PLoS One 9: e94423. 24788455 10.1371/journal.pone.0094423 PMC4006774

[jsr70308-bib-0058] Thomason, M. E. , M. T. Dassanayake , S. Shen , et al. 2013. “Cross‐Hemispheric Functional Connectivity in the Human Fetal Brain.” Science Translational Medicine 5: 173ra24. 10.1126/scitranslmed.3004978 PMC3618956 23427244

[jsr70308-bib-0059] Tokariev, A. , K. Palmu , A. Lano , M. Metsäranta , and S. Vanhatalo . 2012. “Phase Synchrony in the Early Preterm EEG: Development of Methods for Estimating Synchrony in Both Oscillations and Events.” NeuroImage 60: 1562–1573. 22245347 10.1016/j.neuroimage.2011.12.080

[jsr70308-bib-0060] Tokariev, A. , J. A. Roberts , A. Zalesky , et al. 2019. “Large‐Scale Brain Modes Reorganize Between Infant Sleep States and Carry Prognostic Information for Preterms.” Nature Communications 10: 2619. 10.1038/s41467-019-10467-8 PMC6565810 31197175

[jsr70308-bib-0061] Tokariev, A. , M. Videman , J. M. Palva , and S. Vanhatalo . 2016. “Functional Brain Connectivity Develops Rapidly Around Term Age and Changes Between Vigilance States in the Human Newborn.” Cerebral Cortex 26: 4540–4550. 26405053 10.1093/cercor/bhv219

[jsr70308-bib-0062] Tolonen, M. , J. M. Palva , S. Andersson , and S. Vanhatalo . 2007. “Development of the Spontaneous Activity Transients and Ongoing Cortical Activity in Human Preterm Babies.” Neuroscience 145: 997–1006. 17307296 10.1016/j.neuroscience.2006.12.070

[jsr70308-bib-0063] Tomczak, E. , and M. Tomczak . 2014. “The Need to Report Effect Size Estimates Revisited. An Overview of Some Recommended Measures of Effect Size.” Trends in Sport Sciences 1, no. 21: 19–25.

[jsr70308-bib-0064] Uccella, S. , V. Marazzotta , D. Preiti , et al. 2025. “Sleep Architecture Correlates With Neurological and Neurobehavioral Short‐ and Mid‐Term Outcome in a Sample of Very Preterm Infants. A Pilot Study.” Sleep Medicine 131: 106538. 40288251 10.1016/j.sleep.2025.106538

[jsr70308-bib-0065] Uccella, S. , A. Parodi , M. G. Calevo , et al. 2023. “Influence of Isolated Low‐Grade Intracranial Haemorrhages on the Neurodevelopmental Outcome of Infants Born Very Low Birthweight.” Developmental Medicine and Child Neurology 65: 1366–1378. 10.1111/dmcn.15559 36998157

[jsr70308-bib-0066] Uccella, S. , A. Potenzieri , and V. Marazzotta . 2024. Preterm Birth: a Paradigm of Sleep Disruption.

[jsr70308-bib-0067] Uylings, H. B. M. 2006. “Development of the Human Cortex and the Concept of “Critical” or “Sensitive” Periods.” Language Learning 56: 59–90.

[jsr70308-bib-0068] van den Heuvel, M. I. , and M. E. Thomason . 2016. “Functional Connectivity of the Human Brain In Utero.” Trends in Cognitive Sciences 20: 931–939. 27825537 10.1016/j.tics.2016.10.001 PMC5339022

[jsr70308-bib-0069] Vanhatalo, S. , and K. Kaila . 2006. “Development of Neonatal EEG Activity: From Phenomenology to Physiology.” Seminars in Fetal & Neonatal Medicine 11: 471–478. 17018268 10.1016/j.siny.2006.07.008

[jsr70308-bib-0070] Vanhatalo, S. , J. M. Palva , S. Andersson , C. Rivera , J. Voipio , and K. Kaila . 2005. “Slow Endogenous Activity Transients and Developmental Expression of K+‐Cl‐ Cotransporter 2 in the Immature Human Cortex.” European Journal of Neuroscience 22: 2799–2804. 16324114 10.1111/j.1460-9568.2005.04459.x

[jsr70308-bib-0071] Vanhatalo, S. , J. M. Palva , M. D. Holmes , J. W. Miller , J. Voipio , and K. Kaila . 2004. “Infraslow Oscillations Modulate Excitability and Interictal Epileptic Activity in the Human Cortex During Sleep.” Proceedings of the National Academy of Sciences 101: 5053–5057. 10.1073/pnas.0305375101 PMC387372 15044698

[jsr70308-bib-0072] Varley, T. F. , O. Sporns , N. J. Stevenson , et al. 2025. “Emergence of a Synergistic Scaffold in the Brains of Human Infants.” Communications Biology 8: 743. 40360743 10.1038/s42003-025-08082-z PMC12075868

[jsr70308-bib-0073] Vinck, M. , R. Oostenveld , M. van Wingerden , F. Battaglia , and C. M. A. Pennartz . 2011. “An Improved Index of Phase‐Synchronization for Electrophysiological Data in the Presence of Volume‐Conduction, Noise and Sample‐Size Bias.” NeuroImage 55: 1548–1565. 21276857 10.1016/j.neuroimage.2011.01.055

[jsr70308-bib-0074] Wang, S. H. , G. Arnulfo , L. Nobili , et al. 2024. “Neuronal Synchrony and Critical Bistability: Mechanistic Biomarkers for Localizing the Epileptogenic Network.” Epilepsia 65: 2041–2053. 38687176 10.1111/epi.17996

[jsr70308-bib-0075] Wang, S. H. , F. Siebenhühner , G. Arnulfo , et al. 2023. “Critical‐Like Brain Dynamics in a Continuum From Second‐ to First‐Order Phase Transition.” Journal of Neuroscience 43: 7642–7656. 37816599 10.1523/JNEUROSCI.1889-22.2023 PMC10634584

[jsr70308-bib-0076] Whitehead, K. , R. Pressler , and L. Fabrizi . 2017. “Characteristics and Clinical Significance of Delta Brushes in the EEG of Premature Infants.” Clinical Neurophysiology Practice 2: 12–18. 30214965 10.1016/j.cnp.2016.11.002 PMC6123866

[jsr70308-bib-0077] Yrjölä, P. , S. Stjerna , J. M. Palva , S. Vanhatalo , and A. Tokariev . 2022. “Phase‐Based Cortical Synchrony Is Affected by Prematurity.” Cerebral Cortex 32: 2265–2276. 34668522 10.1093/cercor/bhab357 PMC9113310

[jsr70308-bib-0078] Yrjölä, P. , S. Vanhatalo , and A. Tokariev . 2024. “Neuronal Coupling Modes Show Differential Development in the Early Cortical Activity Networks of Human Newborns.” Journal of Neuroscience 44: e1012232024. 38769006 10.1523/JNEUROSCI.1012-23.2024 PMC11211727

[jsr70308-bib-0079] Zimmern, V. 2020. “Why Brain Criticality Is Clinically Relevant: A Scoping Review.” Frontiers in Neural Circuits 14: 54. 32982698 10.3389/fncir.2020.00054 PMC7479292

