# Supplementary material for: Quantifying Cortical Maturational Aspects During Different Vigilance States in Preterm Infants by Advanced EEG Analysis
Source: J Sleep Res. 2026 Feb 8;35(4):e70308. doi: 10.1111/jsr.70308 (PMC13357759; doi:10.1111/jsr.70308)
Supplement: Supplementary file 1 — Data S1: jsr70308‐sup‐0001‐supinfo.docx. Supporting Information. Supplementary Figure 1: No group differences in neuronal dynamics are observed between preterm infants with and without brain lesions. Comparison between non‐lesional and lesional infants of (a) weighted phase‐lag index (wPLI) and (b) bistability index (BiS), shown separately for each vigilance state. Shaded areas represent variability estimated from 100 bootstraps (95th percentile). The second row reports the spectra of effect size (rank‐biserial correlation coefficient, RBC; black) and Benjamini‐Hochberg‐corrected p‐values (red), with the thin dashed line indicating p = 0.05. (c) RBC values for normalised phase–amplitude coupling (nPAC) between non‐lesional and lesional infants for each vigilance state. Positive RBC values indicate higher values in non‐lesional compared to lesional infants (green), while negative values indicate the opposite pattern (pink). More transparent regions correspond to non‐significant differences (p > 0.05, Benjamini‐Hochberg corrected). Supplementary Figure 2: Pairwise Wilcoxon rank‐sum test comparing the PSD across sleep stages. Empty circles indicate p < 0.05 after the Wilcoxon rank‐sum test, while filled circles represent p < 0.05 after Benjamini‐Hochberg (BH) correction. Legend. PSD: power spectral density; QW: quiet wakefulness; SOAS: sleep onset active sleep; AS: active sleep; QS: quiet sleep. Supplementary Figure 3: Pairwise Wilcoxon rank‐sum test comparing the wPLI across sleep stages. Empty circles indicate p < 0.05 after the Wilcoxon rank‐sum test, while filled circles represent p < 0.05 after Benjamini‐Hochberg (BH) correction. Legend. wPLI: weighted phase‐lag index; QW: quiet wakefulness; SOAS: sleep onset active sleep; AS: active sleep; QS: quiet sleep. Supplementary Figure 4: Pairwise Wilcoxon rank‐sum test comparing the BiS across sleep stages. Empty circles indicate p < 0.05 after the Wilcoxon rank‐sum test, while filled circles represent p < 0.05 after Ben [file JSR-35-e70308-s001.docx]

**Quantifying cortical maturational aspects during different vigilance states in preterm infants by advanced EEG analysis**

**Supplementary Material**

# Supplementary Figures

## Supplementary Figure 1


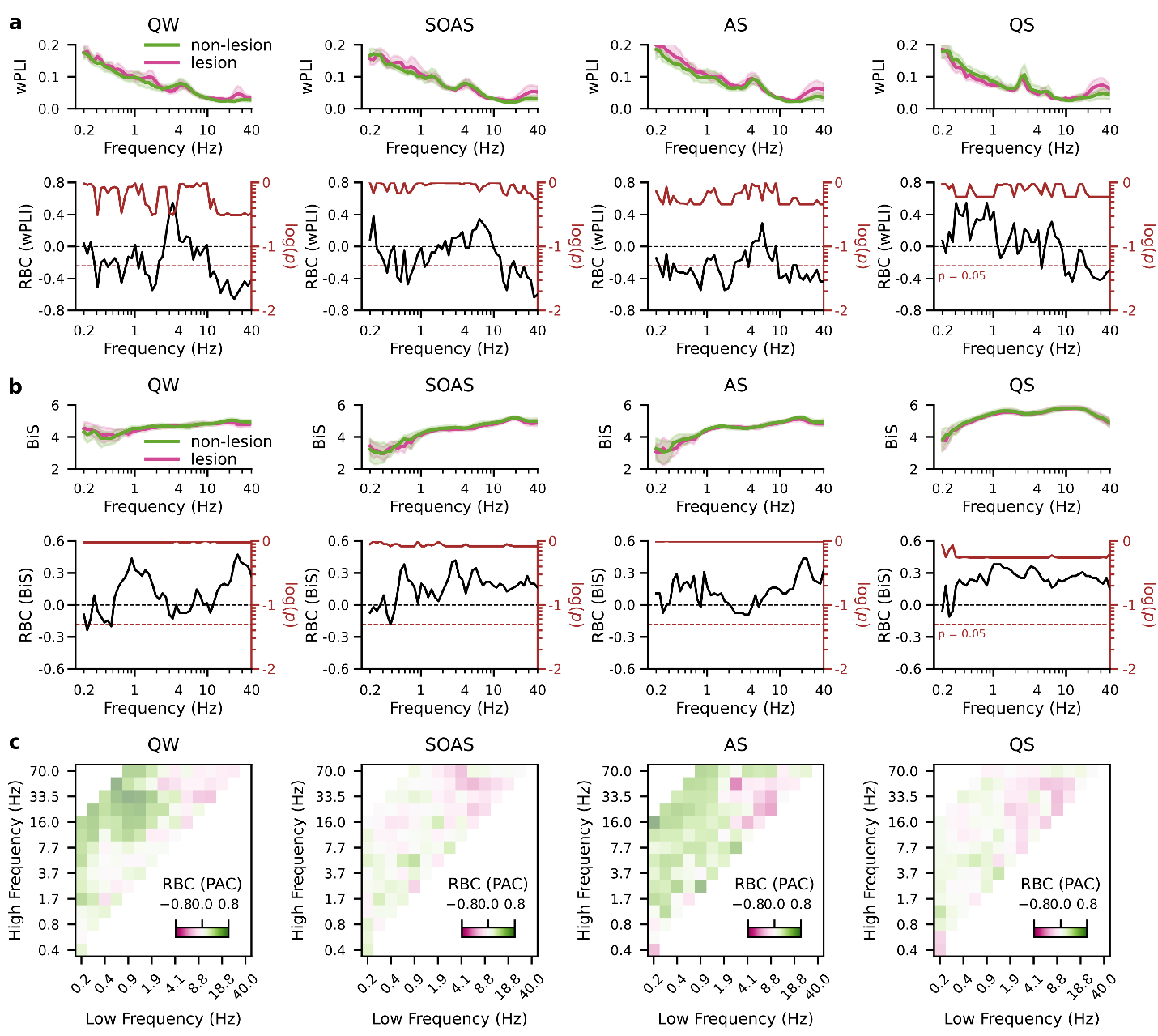


**Supplementary Figure 1: No group differences in neuronal dynamics are observed between preterm infants with and without brain lesions.** Comparison between non-lesional and lesional infants of **(a)** weighted phase-lag index (wPLI) and **(b)** bistability index (BiS), shown separately for each vigilance state. Shaded areas represent variability estimated from 100 bootstraps (95^th^ percentile). The second row reports the spectra of effect size (rank-biserial correlation coefficient, RBC; black) and Benjamini-Hochberg-corrected *p*-values (red), with the thin dashed line indicating *p* = 0.05. **(c)** RBC values for normalized phase–amplitude coupling (nPAC) between non-lesional and lesional infants for each vigilance state. Positive RBC values indicate higher values in non-lesional compared to lesional infants (green), while negative values indicate the opposite pattern (pink). More transparent regions correspond to non-significant differences (*p* > 0.05, Benjamini-Hochberg corrected).

## Supplementary Figure 2


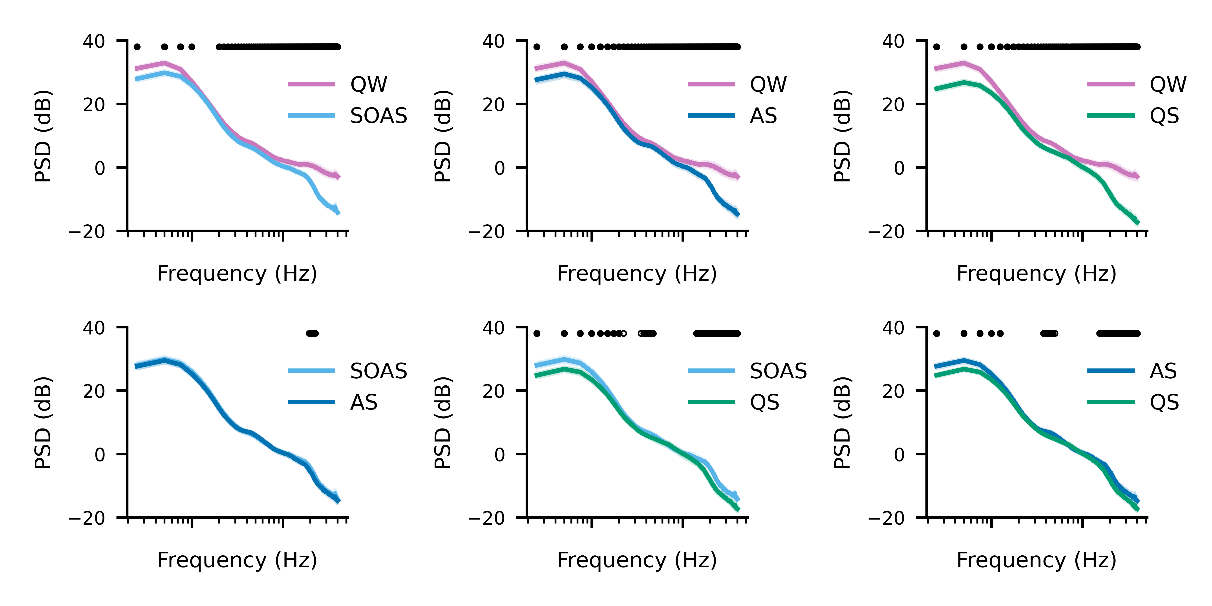


**Supplementary Figure 2:** Pairwise Wilcoxon rank-sum test comparing the PSD across sleep stages. Empty circles indicate *p* < 0.05 after the Wilcoxon rank-sum test, while filled circles represent *p* < 0.05 after Benjamini-Hochberg (BH) correction. **Legend.** PSD: power spectral density; QW: quiet wakefulness; SOAS: sleep onset active sleep; AS: active sleep; QS: quiet sleep.

## Supplementary Figure 3


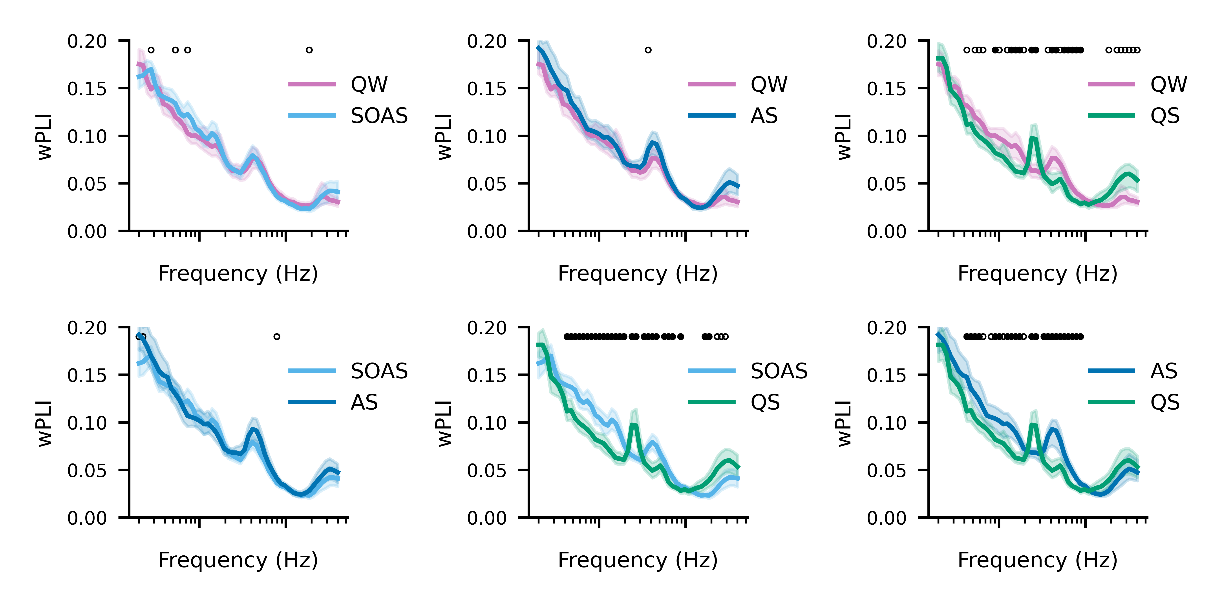


**Supplementary Figure 3:** Pairwise Wilcoxon rank-sum test comparing the wPLI across sleep stages. Empty circles indicate *p* < 0.05 after the Wilcoxon rank-sum test, while filled circles represent *p* < 0.05 after Benjamini-Hochberg (BH) correction. **Legend.** wPLI: weighted phase-lag index; QW: quiet wakefulness; SOAS: sleep onset active sleep; AS: active sleep; QS: quiet sleep.

## Supplementary Figure 4


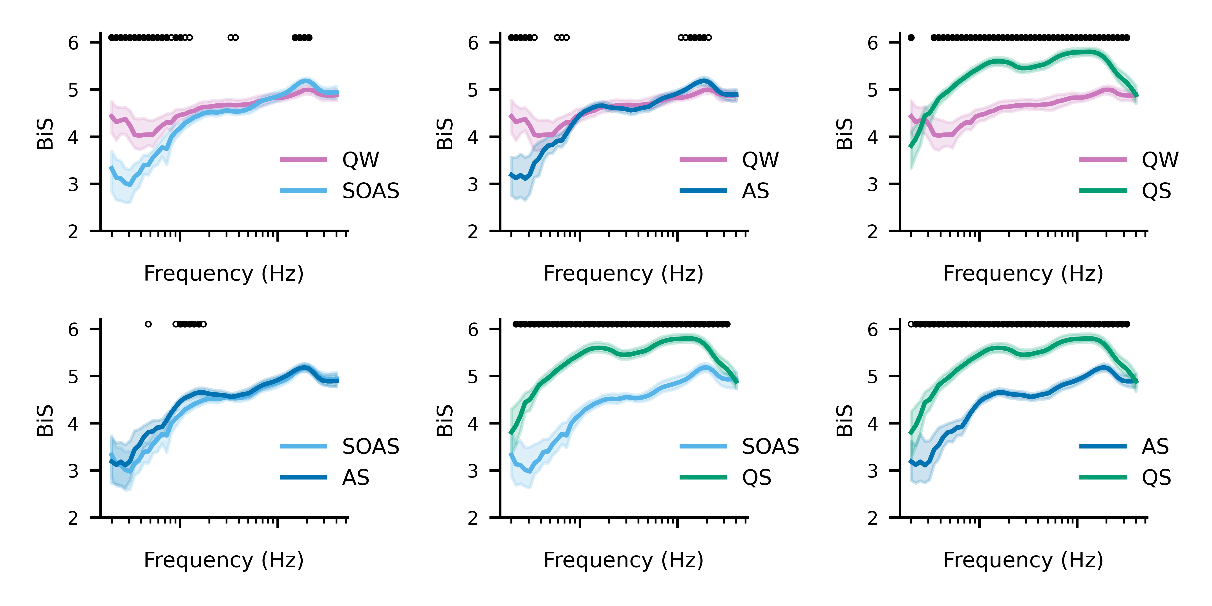


**Supplementary Figure 4:** Pairwise Wilcoxon rank-sum test comparing the BiS across sleep stages. Empty circles indicate *p* < 0.05 after the Wilcoxon rank-sum test, while filled circles represent *p* < 0.05 after Benjamini-Hochberg (BH) correction. **Legend.** BiS: bistability index; QW: quiet wakefulness; SOAS: sleep onset active sleep; AS: active sleep; QS: quiet sleep.

## Supplementary Figure 5


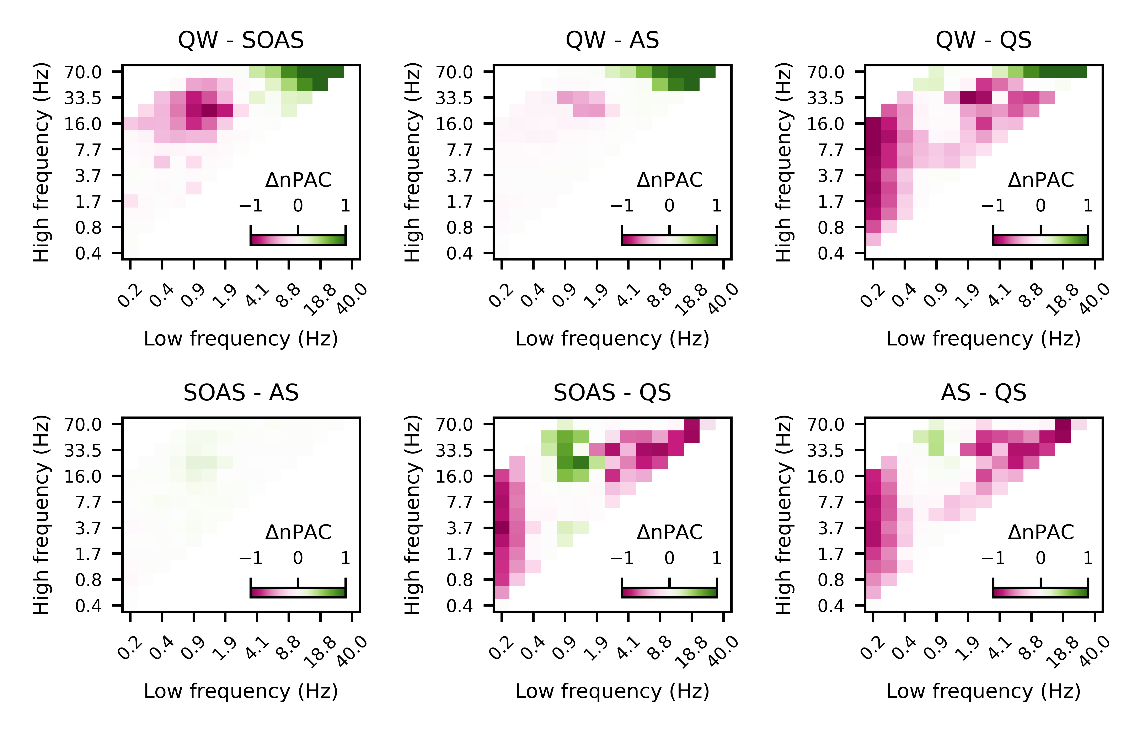


**Supplementary Figure 5:** Difference between each pair of vigilance states nPAC. Alpha shading was applied after pairwise Wilcoxon rank-sum test and Benjamini-Hochberg FDR correction. Regions with less transparency correspond to areas with uncorrected *p* < 0.05. **Legend.** nPAC: normalized phase-amplitude coupling; QW: quiet wakefulness; SOAS: sleep onset active sleep; AS: active sleep; QS: quiet sleep.

## Supplementary Figure 6


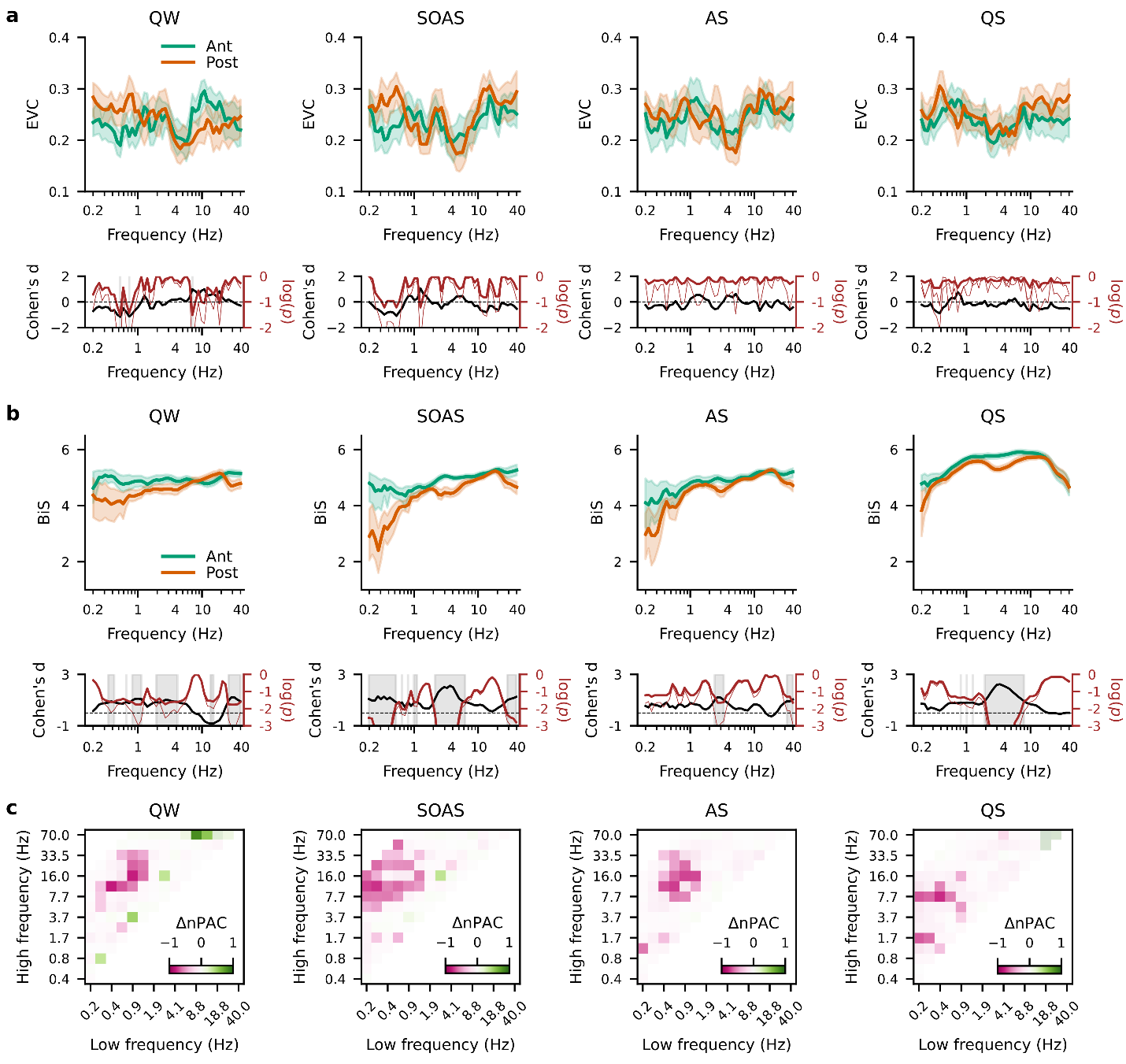


**Supplementary Figure 6:** **Neuronal dynamics differ from occipital (O1-O2) to frontal (Fp1-Fp2) derivations. (a)** Comparison between frontal and occipital eigenvector centrality (EVC) and **(b)** BiS, separately for each vigilance state. The shaded areas represent the variability computed from 100 bootstraps (95^th^ percentile). The second row shows the spectra of effect size (Cohen’s d, black) and *p*-value (reddish) for each frequency. The *p*-value is represented as a thin line (uncorrected) and a thick line (BH-corrected). Grey areas indicate d > 0.8 (large effect) and *p* < 0.05 after Benjamini-Hochberg FDR correction. **(c)** Difference between anterior and posterior nPAC for each vigilance state. Green hues indicate nPAC_Anterior_ > nPAC_Posterior_, whereas pink hues indicate the opposite. Alpha shading was applied after pairwise Wilcoxon rank-sum test and Benjamini-Hochberg FDR correction. Regions with less transparency correspond to areas with d > 0.8 (large effect) and uncorrected *p* < 0.05. **Legend.** EVC: eigenvector centrality; BiS: bistability index; nPAC: normalized phase-amplitude coupling; QW: quiet wakefulness; SOAS: sleep onset active sleep; AS: active sleep; QS: quiet sleep.

# Supplementary Methods

## Synchrony estimates

To estimate first-order synchrony derivatives, we computed eigenvector centrality (EVC) on wPLI matrices, treating them as graphs. In this representation, each channel corresponds to a node, and edges are the wPLI values. EVC quantifies the importance of each node within the network, assigning higher centrality to nodes that are strongly connected to other highly central nodes.

# Supplementary Results

## Model Summary Statistics

To assess the relationship between EEG-derived metrics and postmenstrual age (PMA), we fitted separate mixed linear models for each measure: wPLI (weighted phase-lag index), BiS (bistability index), and local normalized phase-amplitude coupling (nPAC). For each model, we report the residual variance (scale) and the log-likelihood. The scale and log-likelihood values were as follows: for wPLI, scale = 0.2142 and log-likelihood = -293.9711; for BiS, scale = 0.5752 and log-likelihood = -494.8996; for nPAC, scale = 0.1885 and log-likelihood = -261.8939.
